# Translational Models for Glioblastoma: Revolutionizing Drug Development and Personalized Medicine through Clinical Insights

**DOI:** 10.7150/thno.126324

**Published:** 2026-03-17

**Authors:** Gaeun Lee, Yu Jin Kim, Sharon Jeeho Ham, Hyeongjin Ahn, Dayeong Choi, Juhyeong Ha, Jungseub Lee, Hyung-Jin Lee, Jaejoon Lim, Jungho Ahn

**Affiliations:** 1 Department of MetaBioHealth, Sungkyunkwan University, Suwon, Gyeonggi-do 16419, Republic of Korea.; 2 Department of Neurosurgery, Bundang CHA Medical Center, CHA University, Yatap-dong 59, Seongnam 13496, Republic of Korea.; 3 Department of Biomedical Science, College of Life Science, CHA University, Seongnam 13488, Republic of Korea.; 4 Department of Biophysics, Institute of Quantum Biophysics, Sungkyunkwan University, Suwon-si, Gyeonggi-do, Republic of Korea.; 5 Department of Mechanical and Aerospace Engineering, Seoul National University, Gwanak-gu, Seoul-si, Republic of Korea.; 6 Department of Anatomy, College of Medicine, Chungbuk National University, Republic of Korea.

## Abstract

Glioblastoma (GBM) remains one of the most aggressive and treatment resistant brain tumors and continues to present major challenges for effective therapeutic development. The failure of numerous late-stage clinical trials highlights the limited predictive value of conventional preclinical models. Although established cell lines, two-dimensional cultures, and animal models have been extensively employed, existing platforms fail to adequately recapitulate the complex tumor microenvironment, blood brain barrier function, and interpatient heterogeneity that drive therapeutic resistance in GBM.

To address this translational limitation, advanced experimental systems have been developed to more accurately reproduce key features of the human GBM microenvironment through the integration of microengineering approaches, biomaterials, and patient derived cells. This review focuses on recent advances in microfluidic GBM chip models and three-dimensional bioprinted GBM platforms, while also summarizing a broad range of *in vitro* and *in vivo* model systems extending from conventional 2D cultures and organoids to animal-based platforms.

Microfluidic and 3D bioprinting technologies enable controlled reconstruction of critical biophysical and biochemical features of the GBM microenvironment. These systems allow regulation of oxygen availability, drug exposure, and cellular interactions within engineered tumor constructs. As a result, patient specific GBM models with heterogeneous architectures can be generated with improved biological relevance and closer resemblance to *in vivo* tumor behavior. In parallel, *in vivo* systems remain indispensable for capturing systemic pharmacokinetics, host immune responses, and organ level toxicity. Among these, mouse models encompassing syngeneic, xenograft, and genetically engineered mouse models (GEMMs) continue to serve as the foundation of preclinical GBM research, while larger animal models have been explored to better approximate the physiological and immunological features of human disease.

Overall, this review discusses recent advances in GBM model development across microengineered *in vitro* platforms and *in vivo* animal systems and examines their potential to enhance translational accuracy, accelerate drug discovery, and support personalized therapeutic strategies. By emphasizing the technological scope and clinical relevance of these platforms, this work aims to provide practical guidance for the rational selection and integration of GBM model systems in the discovery and optimization of next generation therapeutics.

## Introduction

### Epidemiology/etiology

Glioblastoma (GBM) is the most aggressive form of glioma, a primary malignant brain tumor of the central nervous system (CNS), most commonly arising in the frontal and temporal lobes. It is characterized by rapid proliferation, extensive angiogenesis, and marked resistance to conventional therapies 1, 2.

GBM is the most prevalent and aggressive primary malignant brain tumor, accounting for approximately 15% of all brain tumors and nearly 50% of malignant brain tumors 3. Globally, the incidence of GBM ranges from 3 to 5 cases per 100,000 individuals annually, with a slight male predominance (male-to-female ratio ~1.6:1) 4, 5. The median age at diagnosis is 64 years, and the incidence peaks between 65 and 75 years of age, indicating that age-related genetic and epigenetic alterations may contribute to tumor development. Epidemiological data also suggest variations by ethnicity and geography, with higher rates among Caucasians and populations in urban or industrialized areas, possibly reflecting environmental exposures 6.

The etiology of GBM is multifactorial, involving genetic predisposition, environmental influences, and potential lifestyle factors. Genetic mutations are central to GBM pathogenesis, with somatic alterations in key genes such as TP53, EGFR, and PTEN frequently observed. These mutations disrupt critical pathways regulating cell growth, apoptosis, and DNA repair, driving the aggressive behavior of GBM. Mutations in isocitrate dehydrogenase (IDH) are more common in secondary GBM than in primary GBM and are associated with a better prognosis. Additionally, inherited genetic syndromes such as Li-Fraumeni syndrome, neurofibromatosis type 1, and Turcot syndrome further highlight the role of genetic predisposition in GBM etiology 7.

Environmental factors, particularly exposure to ionizing radiation, have been strongly linked to an elevated risk of GBM. Patients who have undergone therapeutic cranial irradiation for other cancers or those exposed to high levels of environmental radiation have an increased likelihood of developing gliomas later in life 8. Occupational exposure to industrial chemicals, pesticides, and petrochemicals has also been suggested as a potential risk factor, though definitive evidence is still lacking. The role of lifestyle factors, such as smoking, alcohol consumption, and diet, in GBM risk remains unclear, but some studies suggest that poor dietary habits or chronic inflammation related to obesity may contribute to tumor development 9.

Recent studies have also explored the potential role of viral infections in GBM etiology. Human cytomegalovirus (HCMV) has been detected in a significant proportion of GBM tumors, raising questions about its involvement in tumor progression. HCMV may promote genetic instability and immune evasion, although the exact mechanisms and clinical significance remain under investigation 10.

Although extensive research has been conducted on its etiology and risk factors, GBM remains a highly lethal disease with limited prognosis. The median survival time following diagnosis is approximately 12-15 months, with a five-year survival rate of less than 5%. This poor clinical outlook emphasizes the critical need for a deeper understanding epidemiology of GBM and pathogenesis to guide the development of more effective treatment strategies 11.

### Pathogenesis

GBM pathogenesis is characterized by a complex interplay of genetic, molecular, and cellular mechanisms that collectively drive its aggressive nature and resistance to therapy 12. The tumor arises through the accumulation of genetic alterations, including mutations in key regulatory genes, TP53, EGFR, and PTEN, which disrupt essential cellular processes. TP53 mutations impair cell cycle control and DNA damage response, allowing genomic instability and tumor progression. EGFR amplification and mutations, such as the oncogenic EGFR variant III (EGFRvIII) variant, activate downstream signaling pathways, including PI3K/AKT and RAS/RAF/MEK, which promote cell proliferation, invasion, and resistance to apoptosis 13. Loss of PTEN further enhances PI3K/AKT signaling, contributing to tumor cell survival and therapeutic resistance 2.

In addition to these genetic alterations, the molecular pathways involved in GBM pathogenesis include dysregulation of growth factor signaling and cell cycle checkpoints. Aberrant activation of the PI3K/AKT/mTOR pathway supports tumor growth and metabolic adaptation, while the RAS/RAF/MEK/ERK pathway enhances cell proliferation and migration 14. Mutations in IDH1 and IDH2, often observed in secondary GBM, result in the production of the oncometabolite 2-hydroxyglutarate, which alters epigenetic regulation and facilitates tumorigenesis 15. Furthermore, alterations in DNA repair mechanisms, p53 pathway defects and O6-methylguanine-DNA methyltransferase (MGMT) overexpression, contribute to therapeutic resistance by reducing the efficacy of alkylating agents 12.

### GBM microenvironment

The GBM microenvironment is a highly complex and dynamic network comprising tumor cells, non-tumor stromal and immune cells, extracellular matrix (ECM), and soluble factors such as cytokines and metabolites 16. The cellular components include tumor-associated macrophages (TAMs), microglia, astrocytes and endothelial cells, while the non-cellular components consist of ECM elements such as hyaluronic acid (HA), link proteins, tenascins, and proteoglycans like aggrecan, neurocan, versican and phosphacan 17. This environment acts as a key regulator of GBM progression, supporting tumor invasion, proliferation, and therapeutic resistance 18.

**Figure 1:**
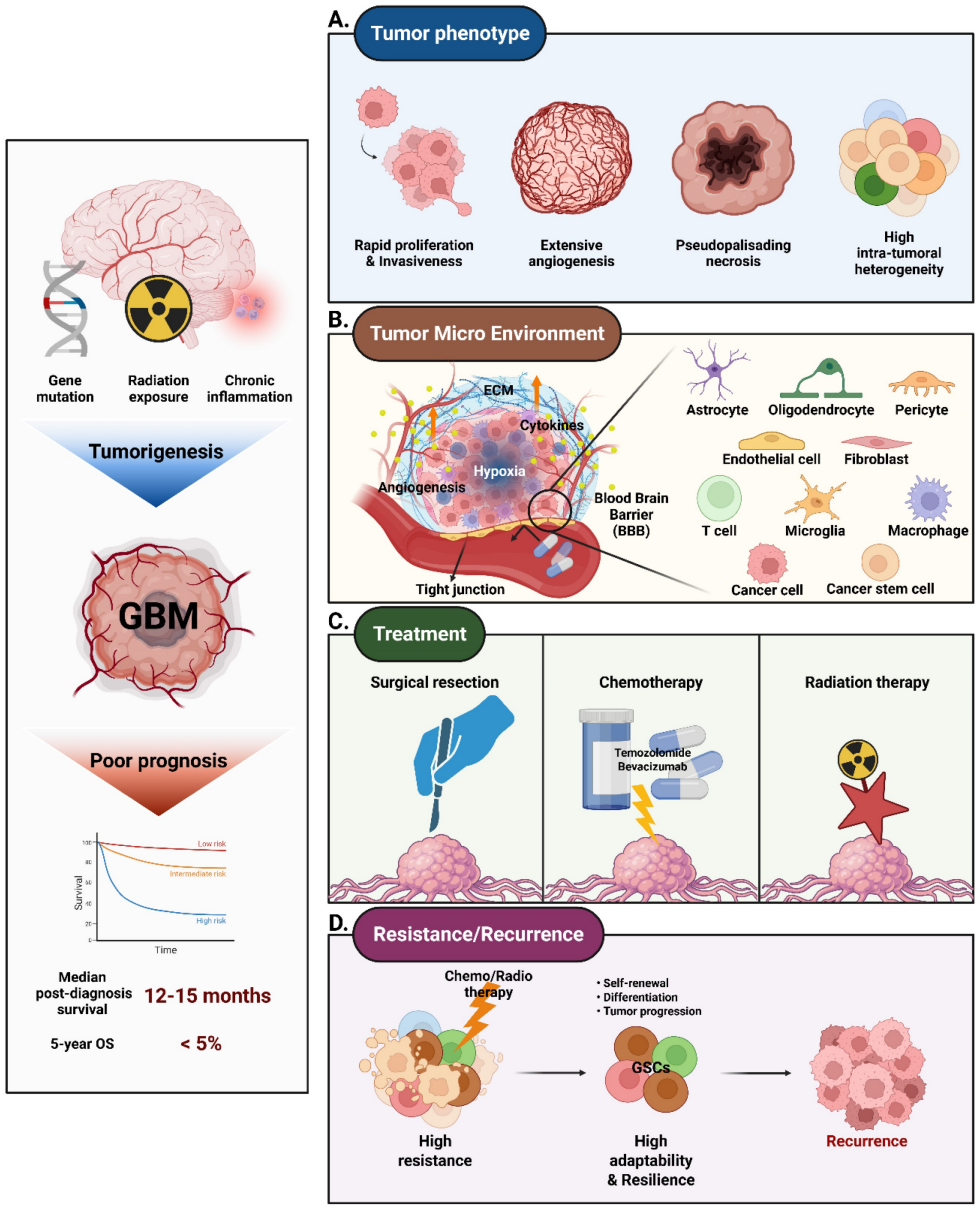
Schematic overview of GBM pathophysiology, TME, and therapeutic challenges.

Furthermore, a hypoxic environment is well known as a key factor in GBM treatment resistance by contributing to angiogenesis, invasion, and survival, and is directly correlated with poor patient prognosis. Changes in partial oxygen tension (pO_2_) are sensed by prolyl-4-hydroxylase 2 (PHD2), which regulates HIFs expression. Under hypoxic conditions, low partial oxygen tension suppresses PHD2, leading to the accumulation of HIF-1α. Dimerization of HIF-1α and HIF-1β induces upregulation of pro-angiogenic genes such as VEGF and fibroblast growth factor, thereby increasing angiogenesis, invasiveness, and tumor metastasis 19. Simultaneously, GBM cells remodel the tumor microenvironment (TME) by secreting exosomes, cytokines, and other soluble factors that influence both surrounding and distant cells within TME. These secreted molecules form physical interactions with stromal and immune cells, reprogramming the behavior of surrounding cells and contributing to therapeutic resistance **(Figure 1A)**. Through these multifaceted interactions, GBM cells exploit the TME to sustain their invasive nature and evade therapeutic interventions. One major obstacle in treating GBM is the blood-brain barrier (BBB), which acts as a highly selective and protective shield. The BBB allows only specific substances, primarily small and lipophilic molecules, to pass through. Effectively delivering anticancer agents to the brain to treat GBM is a significant challenge 16.

The immune cell population in the GBM microenvironment, including TAMs, microglia, and tumor-infiltrating lymphocytes (TILs), plays a crucial role in tumor progression 20. TAMs and microglia, influenced by GBM-derived cytokines and growth factors, often acquire an immunosuppressive phenotype. These cells release anti-inflammatory molecules such as IL-10, transforming growth factor beta (TGF-β), and prostaglandins, promoting an environment conducive to tumor growth 21. Furthermore, TAMs secrete pro-angiogenic factors like vascular endothelial growth factor (VEGF) and IL-8, enhancing the formation of abnormal vasculature that supports tumor survival and dissemination 22. TAMs also contribute to immune evasion by expressing immune checkpoint molecules like PD-L1, which inhibit T cell activation 23.

Astrocytes and endothelial cells significantly contribute to the structural and functional remodeling of GBM TME **(Figure 1B)**. Astrocytes establish gap junctions with tumor cells, facilitating the exchange of metabolic and ionic signals that support tumor proliferation and migration 24, 25. They also secrete cytokines, including IL-6 and CXCL10, which promote tumor invasiveness. Additionally, GBM is characterized by its high vascularity. The tumor develops abnormally structured blood vessels that supply the necessary oxygen and nutrients but are also associated with increased permeability, leading to issues like brain edema. The endothelial cells are central to angiogenesis, forming abnormal and leaky blood vessels within the TME. These vessels provide nutrients and oxygen to the tumor but also create high interstitial pressure, hindering effective drug delivery. The resulting hypoxic environment further promotes tumor aggressiveness.

The ECM within GBM is uniquely adapted to the brain environment and plays an integral role in tumor progression and resistance. Key ECM components, such as HA, tenascins, and proteoglycans, regulate cell adhesion, migration, and invasion. Increased ECM stiffness mechanically promotes GBM proliferation and migration, which is directly correlated with decreased responsiveness to therapy in 3D models. ECM stiffness modulates YAP/TAZ signaling, increasing cytoskeletal tension and upregulating gene expression associated with chemotherapy resistance 26. Specifically, the HA/collagen ratio influences integrin-mediated signaling and drug diffusion. Low-molecular-weight HA promotes tumor invasion, while high-molecular-weight HA can inhibit migration, but may sequester growth factors and limit drug permeability. This balance is often disrupted in GBM 27. Additionally, tenascins and proteoglycans remodel the ECM, creating a structural and biochemical milieu that supports tumor progression and acts as a reservoir for growth factors that drive invasion and angiogenesis 28, 29.

GBM cells interact with the TME through direct and indirect mechanisms to enhance their adaptability and invasiveness. Direct interactions are mediated by gap junctions composed of connexin-43, enabling the exchange of ions, metabolites, and signaling molecules between tumor cells 25. These interactions establish multicellular networks that facilitate coordinated invasion into healthy brain tissue. Indirect interactions involve the release of cytokines, such as VEGF and IL-6 and exosomes that transport bioactive molecules across the BBB.

These mechanisms act in a complex manner in the GBM microenvironment to promote angiogenesis and increase immune evasion, which contributes particularly to treatment resistance. 18. Therefore, the need to develop therapeutic strategies targeting the GBM TME is emphasized to improve patient outcomes.

### GBM recurrence

Recurrent GBM presents a major challenge in cancer treatment due to its aggressive behavior and high resistance to standard therapies 4. Despite initial interventions such as surgery, radiation, and chemotherapy with temozolomide (TMZ), recurrence is almost inevitable **(Figure 1D)** 30. This high recurrence rate is driven by several factors, including tumor heterogeneity, the presence of glioma stem-like cells (GSCs), and the TME 31. GSCs are critical in driving GBM recurrence. These cells possess a heightened resistance to radiation and chemotherapy and contribute to tumor regrowth through their self-renewal capabilities 32. GSCs are recognized as key regulators of tumor initiation and progression. They are closely associated with therapeutic resistance and recurrence by promoting cell survival, angiogenesis, invasion, and tumor dissemination. Studies have highlighted that GSCs promote not only tumor cell survival but also angiogenesis, invasion, and tumor spread, all of which are key factors in recurrence 31. GSCs are characterized by their ability to self-renew and differentiate into various cell types found within the tumor, making them highly adaptable and resilient against treatment 33. GSCs are also known to reside in specialized niches within the TME, such as perivascular regions, where they receive support from the surrounding blood vessels 34. This close association with vasculature not only provides GSCs with necessary nutrients and oxygen but also contributes to their ability to promote angiogenesis through the secretion of pro-angiogenic factors like VEGF 35. This process helps sustain tumor growth and creates a microenvironment that is conducive to recurrence 36. Moreover, GSCs exhibit a high level of plasticity, allowing them to transition between different cellular states in response to therapeutic stress. This plasticity is a key factor in their ability to evade standard treatments, as GSCs can adapt to changing conditions and reinitiate tumor growth even after aggressive therapy 32. GSCs are also highly efficient at repairing DNA damage, which further contributes to their resistance to radiation and chemotherapy 37. The presence of GSCs within the tumor makes it difficult to achieve lasting remission, as these cells can repopulate the tumor and drive recurrence.

The majority of GBM recurrences occur within 2-3 cm of the original tumor site, largely due to the highly infiltrative nature of GBM cells 38, 39. More than 90% of recurrences happen at or near the original tumor location, underscoring the challenge of achieving complete tumor removal even with aggressive surgery 40. The TME, which consists of immune cells, blood vessels, and ECM components, plays a crucial role in supporting tumor cell survival and facilitating regrowth 41. Radiation therapy, though initially effective, can alter the TME in a way that promotes a more aggressive tumor phenotype upon recurrence 42. Changes in the ECM, increased inflammation, and hypoxia contribute to this enhanced aggressiveness by creating a supportive niche for GSCs, enhancing invasive potential, and reducing therapeutic efficacy 43.

Radiation-induced changes in the TME can lead to increased tumor cell invasion, altered cytokine signaling, and chronic inflammation, all of which help create an environment that supports tumor regrowth. These alterations not only promote a more favorable environment for the tumor but also reduce the efficacy of subsequent therapies 44. Additionally, chemotherapeutic stress can induce GBM cells to transdifferentiate into endothelial cells, leading to vascular mimicry that further supports tumor growth and therapy resistance. This plasticity allows GBM cells to adapt under therapeutic pressure and form new tumor-derived blood vessels, ultimately contributing to recurrence 45.

### Clinical presentation

The clinical presentation of GBM is generally related to the functional tumor location in the brain. Patients often present with focal neurological deficits such as motor weakness, sensory disturbances, and language dysfunction, depending on the specific brain regions involved by the tumor 46. For instance, tumors located in the motor cortex commonly lead to weakness or paralysis, while those in the temporal lobe may cause speech difficulties or memory impairments 47. Seizures are a presenting symptom in approximately 25% of patients with newly diagnosed GBM. These episodes tend to be focal but can generalize, and while anticonvulsants are commonly used, they are generally reserved for patients who have experienced these occurrences 48. GBM may also present with non-specific symptoms such as headaches, which are often caused by increased intracranial pressure due to the mass effect of the tumor or obstructive hydrocephalus 49. Cognitive disturbances, personality changes, and mood disorders are common when tumors affect the frontal lobes or other areas involved in executive function 50. Additionally, symptoms such as confusion, memory loss, and fatigue may present in a significant number of patients, complicating early diagnosis since these symptoms are non-specific and can be attributed to various other neurological or systemic conditions 49. The typical imaging characteristics of GBM include a heterogeneously enhancing lesion with a necrotic core, surrounded by a region of peritumoral edema on magnetic resonance imaging (MRI). These tumors are highly infiltrative, extending into the white matter, often involving structures including the corpus callosum 47. This invasive nature contributes significantly to the heterogeneity of clinical presentations, as well as the challenge of achieving complete surgical resection, which in turn impacts prognosis. In some cases, GBM is presented as a thalamic tumor, which carries specific clinical features and challenges due to the deep location within the brain. Patients with thalamic GBM often experience symptoms like hydrocephalus, motor dysfunction, and cognitive changes. These tumors are considered largely unresectable due to their location, and the management is often limited to biopsy, followed by chemoradiation 51. The clinical presentation of GBM is crucial in determining the management approach and expected outcomes.

### Treatment of GBM

Understanding the epidemiology of GBM is crucial for developing strategies for early detection, prevention, and treatment **(Figure 1C)** 52. This understanding also illuminates why GBM treatment is particularly challenging. Despite being the current standard of care, TMZ and radiotherapy exhibit limited efficacy in the treatment of GBM. GBM cells can develop resistance to TMZ, particularly in cases where high MGMT enzyme expression repairs the DNA damage caused by the drug. Although TMZ can cross the BBB, it may not reach sufficient concentrations in the tumor, thereby limiting its effectiveness 53. Additionally, TMZ can cause side effects such as blood cell depletion and immunosuppression, which impact overall patient health. Although radiotherapy remains a mainstay in GBM treatment, it can inadvertently damage adjacent healthy brain tissue, resulting in cognitive impairments, neurological deficits, and long-term complications. These limitations underscore the urgent need for more effective treatment strategies.

#### Surgical resection

Surgical resection represents the foundational step in GBM treatment, aiming to achieve gross total resection (GTR) while preserving critical neurological function. However, complete removal is frequently limited by diffuse tumor infiltration into eloquent brain regions. The use of advanced technologies such as frameless stereotaxy, intraoperative MRI, and 5-aminolevulinic acid (5-ALA) fluorescence has significantly improved the accuracy of tumor localization and resection margins 54.

More recently, near-infrared II (NIR-II, 1000-1700 nm) fluorescence imaging has emerged as a promising adjunct, offering deeper tissue penetration, reduced scattering, and higher signal-to-background ratios compared to visible or NIR-I fluorescence, thereby enabling improved intraoperative visualization of tumor margins 55, 56. In particular, NIR-II-guided imaging has shown potential for delineating infiltrative tumor boundaries beyond the contrast-enhancing regions, addressing a critical limitation of conventional fluorescence-guided surgery in GBM 57. Furthermore, the integration of NIR-II imaging with targeted probes or nanomaterials may enable real-time, molecularly informed surgical guidance, improving resection precision in highly heterogeneous GBM tissues 58.

Preoperative mapping through functional MRI and diffusion tensor imaging (DTI) provides detailed visualization of brain networks, assisting in surgical planning and minimizing postoperative deficits 59, which provide detailed maps of both tumor boundaries and critical brain pathways. These modalities, combined with intraoperative imaging, allow for a tailored surgical approach that maximizes tumor removal while minimizing neurological deficits 60. Despite these innovations, microscopic residual disease remains inevitable, making surgical intervention insufficient on its own.

#### Radiation therapy

Radiation therapy (RT) is a key component of the adjuvant treatment regimen, typically initiated after surgery. The standard approach involves external beam radiation therapy (EBRT) administered in 2 Gy fractions up to a total dose of 60 Gy over six weeks, in conjunction with concurrent TMZ 61. This protocol has been shown to significantly improve overall survival compared to RT alone and is now widely adopted for newly diagnosed GBM. To enhance therapeutic precision and minimize collateral damage to healthy brain tissue, several advanced RT modalities have been developed. Intensity-modulated radiation therapy (IMRT) enables tailored dose distribution to match tumor contours, minimizing exposure to adjacent normal structures 62. Stereotactic radiosurgery (SRS) delivers highly focused, high-dose radiation in a single or few sessions and is often used for recurrent or small lesions. Additionally, proton beam and carbon ion therapies offer improved dose conformity and reduced radiation exposure to non-cancerous brain regions, making them attractive alternatives to conventional photon-based RT 63. Despite these technological advances, radioresistance and tumor recurrence remain significant challenges. Contributing factors include intratumoral heterogeneity, hypoxia-induced cellular adaptations, and activation of DNA repair pathways, all of which diminish RT efficacy 64.

#### Chemotherapy

Chemotherapy plays a central role in GBM treatment by complementing surgical resection and radiotherapy to control tumor progression and extend patient survival. Due to the highly infiltrative nature of GBM, complete surgical excision is rarely possible, making systemic therapy indispensable 65. Among current options, TMZ, an alkylating cytotoxic agent and bevacizumab, a VEGF-targeting anti-angiogenic agent, are most widely used. Despite clinical benefits, treatment resistance remains a major limitation.

TMZ is administered as part of the Stupp regimen, which combines surgery, radiotherapy, and concurrent/adjuvant TMZ, significantly improving survival compared to radiotherapy alone. TMZ acts by methylating DNA, particularly at the O6 position of guanine, triggering mismatch repair cycles and apoptosis 66. TMZ is typically administered as part of the Stupp protocol, which involves maximal safe surgical resection, followed by radiotherapy combined with daily TMZ for six weeks, and subsequent adjuvant TMZ cycles. This regimen has been shown to significantly improve median survival compared to radiotherapy alone, increasing the likelihood of patients surviving beyond two years 67. However, the long-term efficacy of TMZ is often limited by acquired resistance mechanisms. One of the primary causes is the expression of MGMT, which repairs TMZ-induced DNA lesions and prevents apoptosis. High MGMT activity is associated with poor treatment response, whereas MGMT promoter methylation, which silences gene expression, is correlated with increased TMZ sensitivity and improved survival outcomes 68. In addition to MGMT-related resistance, defects in the mismatch repair (MMR) pathway and enhanced base excision repair (BER) allow tumor cells to tolerate or correct TMZ-induced DNA damage, further diminishing its therapeutic efficacy 69. GSCs characterized by their self-renewal capacity and resistance to conventional therapies, contribute significantly to treatment failure and tumor recurrence. These cells can survive cytotoxic stress and repopulate the tumor following treatment. To address TMZ resistance, several adjunct strategies are under investigation. These include MGMT inhibitors, such as O6-benzylguanine and lomeguatrib, although their clinical application is limited by systemic toxicity 70. In addition, poly ADP-ribose polymerase (PARP) inhibitors have been investigated to block DNA repair pathways, thereby enhancing TMZ-induced cytotoxicity 71. Targeting the TME, particularly angiogenesis, offers an additional therapeutic avenue. Bevacizumab, a monoclonal antibody against vascular endothelial growth factor (VEGF), reduces neovascularization and alleviates peritumoral edema, thereby improving progression-free survival and reducing corticosteroid dependence 72. However, its impact on overall survival remains limited. To overcome VEGF inhibition, GBM cells activate alternative pro-angiogenic pathways, notably those mediated by PDGF and FGF. They also enhance their invasive capacity and undergo metabolic reprogramming to survive under hypoxic conditions 73. These limitations have driven interest in multi-targeted kinase inhibitors that simultaneously block multiple angiogenic signals. Combination strategies integrating anti-angiogenic therapy with immune checkpoint blockade or metabolic pathway inhibitors are also under evaluation to overcome resistance and improve therapeutic outcomes 74.

## *In vitro* GBM models

### Conventional 2D models

In GBM research, various 2D cell models are extensively employed, each offering distinct advantages for investigating tumor biology, therapeutic responses, and genetic alterations 75. These models include established cell lines, primary patient-derived cells, and advanced genetically engineered models, with co-culture systems emerging to better replicate the TME. Among the most widely used are immortalized patient-derived GBM cell lines, which offer cost-efficiency and ease of use in a range of experimental settings 76. Prominent examples include U87MG, T98G, A172 and LN229 75.

The U87MG cell line, a central model of GBM research for decades, is favored for its ease of culture and rapid proliferation 77. It is commonly applied in drug screening and signal transduction studies. However, long-term culture has led to significant genetic drift from the original tumor, limiting its ability to fully represent GBM biology 78. T98G, known for its resistance to both chemotherapy and radiotherapy, serves as a robust model for studying treatment resistance mechanisms 79. It exhibits increased DNA repair capacity, including elevated DNA-PKcs activity, which contributes to its radioresistant phenotype 80. A172 harbors mutations in the tumor suppressor gene *TP53* and is primarily used to study cell cycle regulation, DNA repair, and tumor progression under p53-deficient conditions 81. Similarly, LN229, another *TP53*-mutant line, is often employed in apoptosis-related research and in evaluating pro-apoptotic therapeutic agents 82. Beyond established lines, primary patient-derived GBM cells offer greater clinical relevance. Isolated directly from tumor tissue, these cells retain patient-specific genetic heterogeneity, invasive capacity, and cancer stem-like traits when cultured under 2D conditions 83.

In particular, GSCs provide a more accurate model of GBM, reflecting key features such as self-renewal, therapy resistance, and tumorigenic potential. These cells are essential for studying disease progression, recurrence, and the development of therapies targeting tumor-initiating populations. GSC-based models—including neurospheres, organoids, and patient-derived xenografts—recapitulate the TME and are indispensable tools in preclinical research. However, their use presents challenges such as slow proliferation and potential loss of tumor characteristics during extended culture 84.

### Microfluidic - 3D bioprinting fabrication

The development of GBM-on-a-chip platforms starts with the careful definition of components necessary to replicate the GBM TME. Key design considerations include the selection of pathophysiologically relevant cell types, the recreation of spatial tissue architecture, and the incorporation of physicochemical factors such as biochemical gradients, ECM stiffness, and shear stress. Beyond their structural role, dynamic flow and shear stress in GBM-on-a-chip platforms function as critical biological regulators of tumor behavior and drug transport. Interstitial flow within microfluidic GBM models has been shown to actively promote glioma cell migration and invasion by generating convective chemokine gradients that induce CXCR4/CXCL12-dependent autologous chemotaxis, resulting in enhanced directional motility within three-dimensional extracellular matrices 104. In parallel, shear stress applied to vascularized or BBB-like GBM-on-a-chip systems modulates endothelial cell alignment, junctional integrity, and barrier permeability, thereby directly influencing therapeutic penetration across tumor-associated vasculature and BBB interfaces 105. By enabling precise and reproducible control of these biomechanical and transport cues, microfluidic GBM-on-a-chip platforms provide a mechanistically grounded framework to interrogate glioma invasion dynamics and treatment delivery that cannot be captured in static *in vitro* cultures.

These criteria guide the engineering of microfluidic platforms through conventional techniques such as photolithography, replica molding, and soft lithography, with more recent advances incorporating high-resolution 3D bioprinting 106. In soft lithography-based approaches, microchannel templates are first patterned on silicon wafers using photolithography. Polydimethylsiloxane (PDMS) is then poured onto these molds and cured to create flexible, transparent microstructures. Once bonded to glass or other polymeric substrates, these structures form closed microfluidic channels capable of simulating tissue-tissue interfaces, fluidic shear, and spatial compartmentalization characteristic of the brain tumor environment.

More recently, 3D bioprinting has emerged as a transformative tool in GBM chip development by enabling the layer-by-layer deposition of biofunctional materials and living cells with high spatial precision. This technique allows for the incorporation of diverse cell populations including GBM cells, endothelial cells, astrocytes, fibroblasts, and immune cells into ECM-mimetic hydrogels such as gelatin methacrylate (GelMA), alginate, and fibrin. By replicating the structural and biochemical complexity of native GBM tissues, bioprinting supports physiologically relevant mechanical environments essential for tumor progression and therapeutic evaluation 107, 108. In another approach, a hybrid platform was fabricated by combining 3D bioprinted GBM and vascular compartments with a PDMS-based microfluidic channel network. Bioinks tailored to each cell type GelMA-alginate for tumor cells and GelMA-fibrin for endothelial cells enabled spatially organized tissue construction. This system not only permitted the application of dynamic shear stress via perfusion but also supported long-term monitoring of tumor spheroid behavior. When subjected to simulated microgravity conditions, the platform revealed that biomechanical unloading markedly suppressed spheroid formation and altered cellular morphology and signaling, highlighting the importance of physical forces in GBM biology and treatment resistance **(Figure 3A)** 102. In comparison to other advanced *in vitro* GBM models, such as patient-derived organoids, microfluidic and 3D bioprinting-based platforms offer distinct advantages in controllability and standardization. While organoid models preserve patient-specific genetic and phenotypic features and recapitulate key histopathological traits, they provide limited control over spatiotemporal microenvironmental cues, as gradients of oxygen, nutrients, and therapeutics arise primarily through diffusion. In contrast, microfluidic systems enable reproducible regulation of biochemical and biomechanical parameters, including perfusion-driven transport, shear stress, and vascular barrier function, while 3D bioprinting allows deterministic control over tissue architecture and ECM composition. Together, these approaches complement organoid models by integrating patient-derived heterogeneity with programmable microenvironmental control 109.

**Figure 2:**
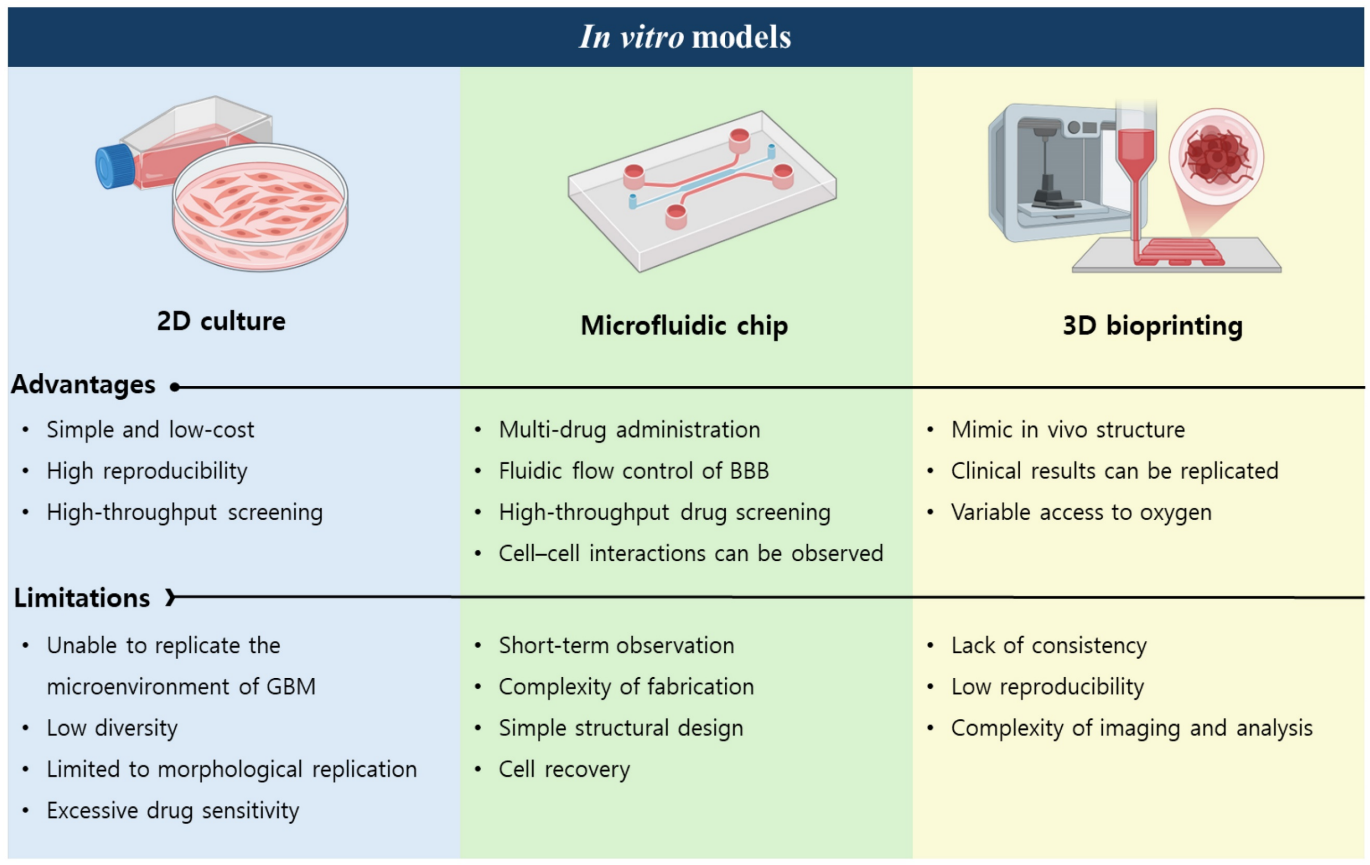
Comparison of *in vitro* models for biomedical research.

The convergence of microfluidic engineering and 3D bioprinting has thus enabled the creation of advanced GBM-on-a-chip systems capable of recapitulating patient-specific tumor heterogeneity, vasculature, and drug response. These hybrid models provide a physiologically relevant framework for dissecting tumor-stroma interactions and conducting high-throughput therapeutic screening, offering a crucial bridge between oversimplified 2D cultures and complex *in vivo* systems **(Figure 2)**.

### Introduction of GBM-on-a-chip

To develop biomimetic three-dimensional tumor models that accurately replicate the complexity of tumor pathophysiology, it is essential to incorporate the critical components of the TME. Among these, oxygen tension serves as a key regulator of cellular behavior, profoundly influencing processes such as proliferation, migration, and therapeutic resistance. This aspect is particularly significant in the case of GBM, the most common and aggressive form of primary malignant brain tumor, which presents a formidable therapeutic challenge due to its profoundly immunosuppressive microenvironment.

Conventional *in vitro* tumor models are typically cultured under normoxic conditions, approximating atmospheric oxygen levels, which do not faithfully reflect the dynamic and heterogeneous hypoxic conditions present within tumors *in vivo*. As a result, these models may produce findings that are not fully representative of the true biological behavior of tumors, potentially leading to inaccurate interpretations 41. This limitation becomes especially critical when modeling metastasis. To accurately replicate the process of intravasation and dissemination observed *in vivo*, tumor cells migrating from the primary site must respond to a variety of microenvironmental cues, including fluctuating oxygen gradients.

**Figure 3:**
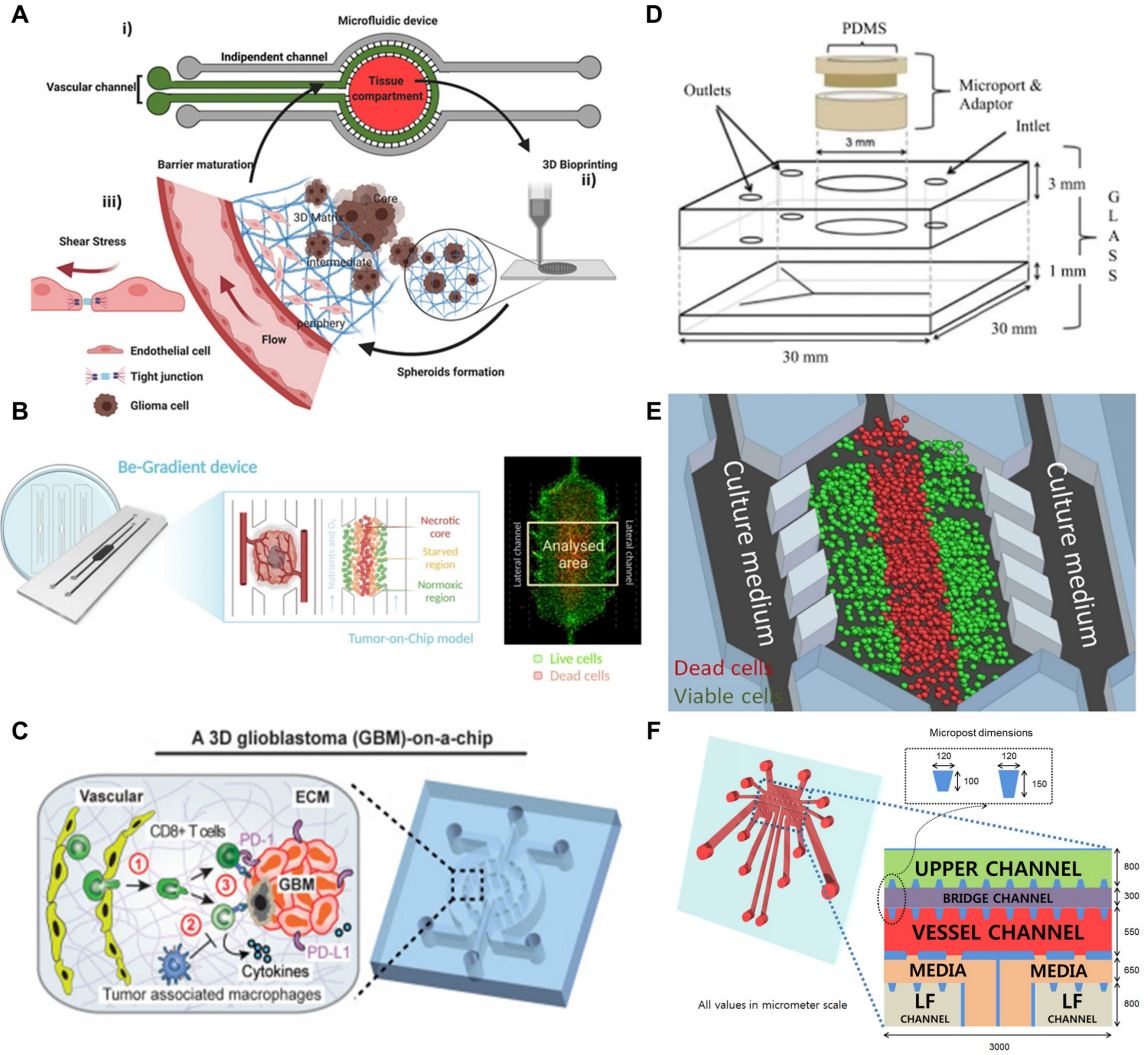
** Microfluidic models of GBM. (A)** Hybrid GBM-on-a-chip platform integrating 3D bioprinted tumor-vascular compartments with microfluidic perfusion. (i) Schematic of the microfluidic device featuring separate vascular and tissue compartments allowing independent control and perfusion. (ii) 3D bioprinting of GBM and endothelial cell compartments using GelMA-alginate and GelMA-fibrin bioinks, respectively. (iii) Cross-sectional illustration showing GBM spheroid formation within a 3D matrix under perfused conditions and shear stress. Reproduced from under 102 an open-access license, © 2021 Adv. Ther. **(B)** Graphical abstract illustrating the design of a GBM-on-a-chip platform that establishes hypoxic gradients to mimic the oxygen-deficient TME of GBM in the human brain. Live and dead cells are fluorescently labeled in green and red. Reproduced from under 110 an open-access license, © 2024 Cell Death Dis. **(C)** A schematic illustration of a GBM-on-a-chip platform representing the interaction between patient-derived GBM cells and primary human immune cells under immunosuppressive tumor microenvironmental conditions. Reproduced from under 91 an open-access license, © 2020 eLife. **(D)** *Ex vivo* culture of patient-derived GBM tissue within a microfluidic device. Schematic of a syringe pump-driven microfluidic chip enabling continuous tissue perfusion. Reproduced from 90 an open-access license, © 2019 Transl. Oncol. **(E)** A GBM-on-a-chip system incorporating a stiffness gradient on a fibronectin-conjugated polyacrylamide hydrogel substrate. (i) A schematic diagram illustrating the design of the GBM-on-a-chip platform with a stiffness gradient. (ii) Fluorescent imaging of U251 MG cells and HCT-116 cells, with live and dead cells labeled in green and red, respectively. Reproduced from under 111 an open-access license, © 2016 Sci. Rep. **(F)** Microfluidic platforms to demonstrate angiogenesis. Schematic illustration of a metastasis-on-a-chip model designed to recapitulate tumor-induced angiogenesis. Reproduced with permission from Ref. 112 © 2014 Biomicrofluidics.

In the context of GBM, the intricate interplay among hypoxia, immune evasion mechanisms, and the ECM creates a highly resilient TME that substantially diminishes the efficacy of conventional therapeutic modalities, including surgical resection, radiotherapy, and TMZ chemotherapy. Although emerging strategies such as immunotherapy and the integration of bioengineering technologies offer promising therapeutic avenues, their overall impact remains substantially constrained by the ability of the tumor to suppress and modulate immune responses. Microfluidic-based platforms present a promising solution to these challenges. These systems are capable of generating precisely controlled oxygen gradients with high spatial and temporal resolution, thereby enabling the creation of more physiologically relevant tumor models 113. A microfluidic GBM model can recreate physiologically relevant oxygen gradients by employing gas-impermeable materials. This design enables the formation of a hypoxic core, a nutrient-deprived intermediate zone, and an oxygen-rich periphery through controlled oxygen and nutrient delivery via side channels **(Figure 3B)** 113. By better simulating the oxygen dynamics of the native TME, microfluidic models provide valuable insights into how fluctuating oxygen levels and other microenvironmental factors influence critical tumor behaviors, including progression, invasion, and therapeutic resistance. This approach is especially effective for investigating complex malignancies like GBM, in which the TME plays a critical role in driving disease progression and shaping treatment response. TME is composed of diverse cellular constituents, including GSCs, endothelial cells, microglia, astrocytes, and neurons, as well as non-cellular components such as the ECM, dynamic hypoxia gradients, and various soluble signaling molecules (**Figure 3C**) 114. As an example, a patient-specific GBM-on-a-chip platform replicates the complex and immunosuppressive microenvironment of GBM. This platform enables real-time analysis of subtype-dependent immunotherapeutic responses, including differences in T cell infiltration and macrophage polarization. Overall, the platform serves as a physiologically relevant tool to study dynamic tumor-immune interactions and supports the personalized screening of immunotherapeutic strategies 91. Taken together, these elements collectively drive the aggressive phenotype of GBM and contribute to its notorious therapeutic resistance.

Therefore, to accurately model GBM *in vitro*, it is imperative to closely recapitulate both the cellular and non-cellular components of the TME. Microfluidic systems, with their capacity to precisely control environmental variables, represent an ideal platform for constructing such advanced and physiologically relevant GBM models.

### Microfluidic GBM models

GBM remains a highly aggressive malignancy with poor prognosis, regardless of the treatment approach. To better study GBM biology under physiologically relevant conditions, microfluidic platforms capable of maintaining human GBM tissues *ex vivo* have been developed (**Figure 3D**) 90. Although still in the early stages, these technologies offer promising opportunities for improving preclinical modeling. Larger patient-derived studies are expected to further validate their utility in predicting treatment responses and in developing more effective therapeutic strategies 90. Among recent innovations, a microfluidic chip integrating a controllable stiffness gradient with orthogonal chemical stimulation has been introduced to investigate GBM cell behavior 115. In this platform, a fibronectin-conjugated polyacrylamide hydrogel generates a longitudinal stiffness gradient, while chemical stimulation is delivered laterally via diffusion. Studies with U87MG cells demonstrated that increased stiffness promotes chemotaxis, and that the presence of an epidermal growth factor (EGF) gradient further accelerates migration. These results underscore the critical influence of biophysical and biochemical gradients in regulating GBM cell motility.

Efforts to recapitulate the perivascular niche (PVN) of GBM have led to the development of microvasculature-on-a-chip systems. These models recreate a microenvironment where brain tumor stem-like cells (BTSCs) preferentially localize near microvessels and exhibit distinct behavioral phenotypes either quiescent or invasive depending on their molecular subtype. Single-cell transcriptomic analyses revealed correlations between microvessel proximity and proneural or mesenchymal subtypes, effectively capturing patient-specific tumor heterogeneity 116. Building on these approaches, a three-dimensional organotypic microfluidic platform has been constructed by integrating hydrogel-based biomaterials with engineered microvascular networks 117. This system successfully maintained GSCs invasive morphology, proliferation, and stemness, mirroring *in vivo* observations. Moreover, the model identified the CXCL12-CXCR4 signaling axis as a critical mediator of GSCs invasion. Inhibition of this pathway with AMD3100 significantly reduced invasive behavior, highlighting the potential of this model for drug screening applications 118. Microfluidic technology has enabled more realistic modeling of the GBM microenvironment.

Microfluidic platforms have also been instrumental in modeling the effects of hypoxia within the GBM microenvironment. One model precisely controlled oxygen and nutrient levels to replicate the formation of pseudopalisades, wherein dense clusters of tumor cells migrate away from hypoxic and nutrient-deprived zones caused by vascular occlusion 119, 120. This system enabled real-time observation of hypoxia-driven migration, providing insights into tumor adaptation under metabolic stress 121.

Expanding upon this, another microfluidic system generated continuous gradients of oxygen and nutrients within a hydrogel matrix, establishing distinct tumor zones comprising normoxic, hypoxic, and necrotic regions. This model facilitated dynamic monitoring of processes such as proliferation, apoptosis, and reactive oxygen species (ROS) production, while also serving as a platform for evaluating the penetration and efficacy of therapeutics, particularly those targeting hypoxic tumor areas (**Figure 3E**) 111. In addition, metastasis-on-a-chip systems have been developed to study tumor-endothelial interactions by forming perfusable microvessels that mimic natural vasculature (**Figure 3F**). These models enable investigation of key processes in cancer progression, including angiogenesis and tumor cell migration across endothelial barriers, and offer valuable tools for testing the efficacy of anti-angiogenic therapies 112. Collectively, microfluidic GBM models provide a highly controlled environment for replicating key physiological and pathophysiological features of GBM. By enabling precise manipulation of microenvironmental conditions such as stiffness, chemical gradients, oxygen availability, and vascular interactions, these platforms are poised to significantly enhance the development of targeted therapies and personalized treatment strategies for GBM.

Building on these advances in microfluidic GBM modeling, patient-derived microengineered platforms have begun to extend these approaches toward clinically relevant translation. In the submitted manuscript, a microengineered GBM model was developed that integrates a 3D cellular network composed of normal human astrocytes and primary tumor cells isolated from surgically resected GBM tissue. Within a defined microfluidic environment, patient-derived GBM cells and astrocytes self-organize into interconnected 3D structures, enabling physiologically relevant investigation of patient-specific cellular interactions and therapeutic responses. Comparison of chip-based tumor responses with clinical outcomes following conventional chemotherapy revealed a strong correspondence between *in vitro* phenotypes and patient prognosis. Specifically, the chip derived from patient A exhibited a markedly disrupted barrier and minimal responsiveness to high-dose TMZ, consistent with the shortest progression-free survival and overall survival. In contrast, the patient B derived chip maintained robust barrier integrity and demonstrated pronounced TMZ sensitivity, aligning with the most favorable clinical outcome. Collectively, these observations indicate that patient-derived microfluidic GBM models can capture clinically meaningful, patient-specific therapeutic responses and highlight their potential as functional *in vitro* platforms for personalized therapy evaluation 122.

### 3D bioprinting GBM models

**Figure 4:**
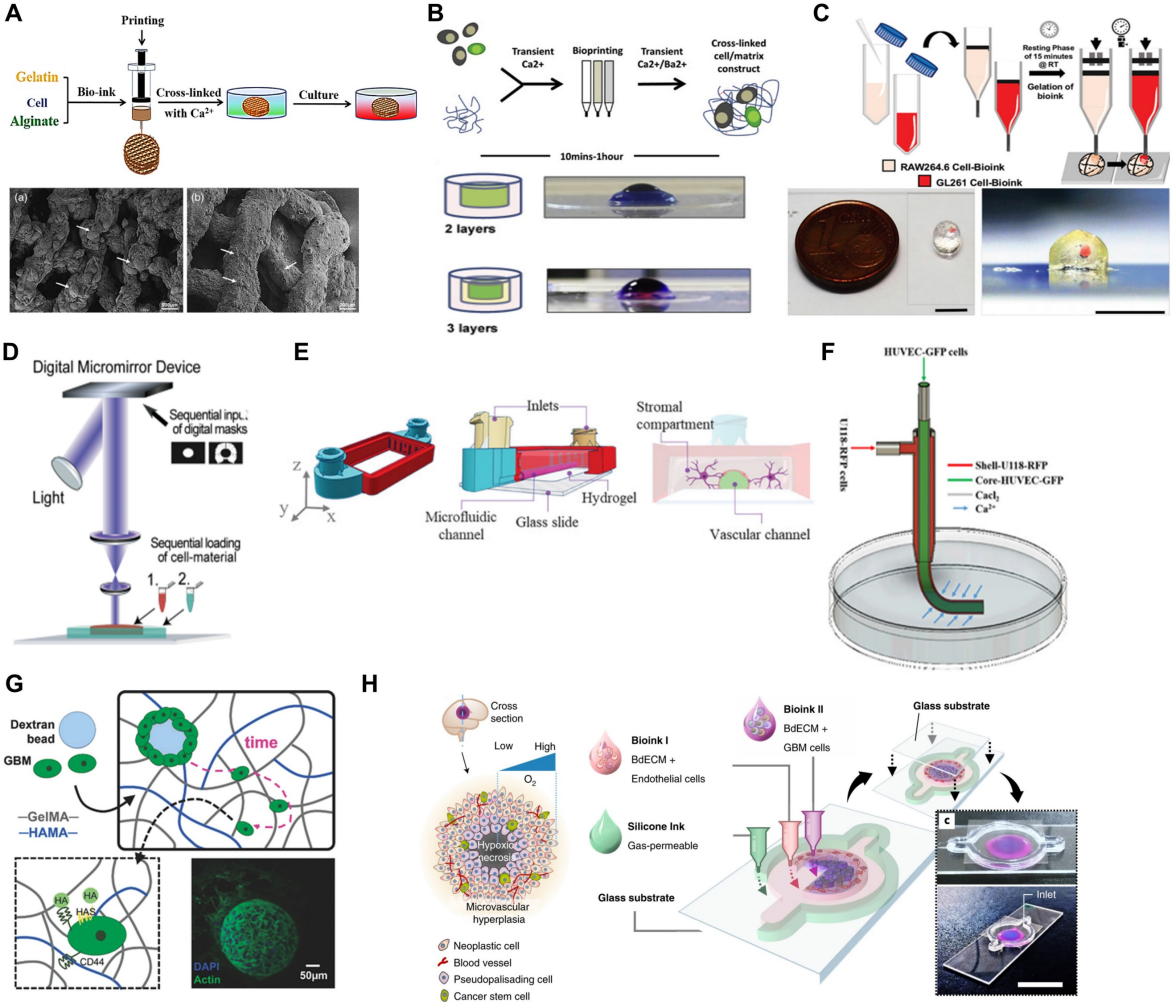
** 3D Bioprinting approaches for modeling the GBM microenvironment. (A)** 3D bioprinting and SEM characterization of U118 cell-laden hydrogel scaffolds, illustrating the formation and sprouting of GSC23 and U118 spheroids (white arrows) on day 15. Reproduced with permission from Ref. 123 © 2020 J. Biomed. Mater. Res. Part A **(B)** Schematic workflow of alginate-based extrusion bioprinting using transient Ca²⁺ crosslinking and representative images of multi-layered constructs consisting of a cancer cell core encapsulated by stromal cell layers. Reproduced with permission from Ref 124 © 2020 Adv. Biol. Regul. **(C)** Schematic of the fabrication process for cell-laden mini-brain constructs and corresponding images showing the spatial organization of GL261 GBM cells (red) and RAW264.7 macrophages within the bioprinted model (scale bar = 5 mm). Reproduced with permission from Ref. 125 © 2019 Adv. Mater. **(D)** A schematic representation of the digital light processing bioprinting method. Reproduced with permission from Ref 98. **(E)** A schematic representation of stereolithography (SLA) bioprinting method. Reproduced from under 126 an open-access license, © 2024 Adv. Healthcare Mater. **(F)** Coaxially bioprinted GBM microenvironment showing the printing process and corresponding bright-field and fluorescence images of U118-RFP GBM cells in the outer shell and HUVEC-GFP endothelial cells embedded in the hydrogel core. Reproduced from under 95 an open-access license, © 2021 Front. Bioeng. Biotechnol. **(G)** A schematic illustration of GBM cell behavior within a GelMA and HAMA-based hydrogel encapsulating a dextran bead. Reproduced with permission from Ref 127. © 2018 Colloids Surf. **(H)** Schematic illustration of a cross-sectional view of concentric-ring GBM-on-a-chip model integrating patient-derived GBM and endothelial cells. Reproduced with permission from Ref 103 © 2019 Nat. Biomed. Eng.

Bioprinting, a specialized form of 3D printing, enables the spatially controlled deposition of bioinks containing cells and biomaterials to engineer complex tissue-like constructs. In GBM research, bioprinting facilitates the development of *in vitro* models that better capture tumor heterogeneity, including interactions with the ECM, immune cells, and vasculature 118, 123. Techniques such as extrusion-based bioprinting (EBB), digital light processing (DLP), and stereolithography (SLA) have been employed to construct physiologically relevant GBM models (**Figure 4A**).

EBB remains the most frequently used technique for fabricating GBM constructs 128. In this method, cell-laden hydrogels are extruded layer-by-layer to form 3D architectures 129, 130. This technique supports high cell densities and is particularly suitable for co-printing GBM cells with stromal elements, such as endothelial cells and fibroblasts, within hydrogels 131, 132.

EBB has enabled the creation of models incorporating GSCs such as G144, G166, and G7 alongside endothelial cells, simulating tumor-vascular interactions and recapitulating chemoresistance mechanisms not observed in 2D cultures 124 (**Figure 4B**). The co-culture of GSCs with endothelial cells in a 3D bioprinted model allows researchers to observe how these cells interact, proliferate, and develop resistance to chemotherapeutic agents like TMZ. These processes are not typically observed in 2D culture systems. The 3D bioprinting model more accurately mimics the complex cell interactions and drug resistance mechanisms occurring in the TME 133. The GAMs significantly contribute to GBM progression and invasiveness, yet their interaction with GBM cells remains poorly understood. A novel 3D-bioprinted mini-brain model, incorporating both GBM cells and macrophages, has been developed to study this crosstalk and test targeted therapies (**Figure 4C**). The model shows that GBM cells recruit and polarize macrophages into a GAM-specific phenotype, mirroring patient data. Additionally, GAMs enhance tumor growth and invasion, while therapeutic interventions targeting their interaction reduce tumor progression and improve chemotherapy sensitivity. This platform offers a valuable tool for advancing tumor biology research and evaluating new treatments 125. In another study, researchers developed an advanced GBM model using a fibrin-based bioink incorporating patient-derived GBM cells, stromal cells, and perfusable vascular networks 101. This model effectively mimics the TME, including cellular heterogeneity and vascular architecture, allowing detailed studies on tumor growth, invasion, and drug resistance. By incorporating patient-specific features and dynamic perfusion, the platform bridges the gap between traditional 2D cultures and *in vivo* models, offering a robust tool for personalized therapy screening and drug discovery.

DLP and SLA are other key technologies used in bioprinting GBM models 134. These methods rely on light to cure bioinks layer by layer, allowing for the creation of highly detailed structures with region-specific properties. DLP and SLA are especially useful in generating GBM models with varying stiffnesses, which is critical for studying the mechanical influences on tumor growth and drug response (**Figure 4D**) 98, 135. In GBM, the tumor core is often stiffer than the surrounding brain tissue, and this difference in stiffness affects cell behavior. DLP bioprinting has been used to create multi-stiffness GBM models that replicate this heterogeneity, enabling the study of how different stiffness environments influence glioma invasion, stemness, and resistance to therapies 136, 137.

For instance, a tri-regional GBM model was developed using DLP-based bioprinting, consisting of tumor, ECM and endothelial regions. By adjusting the stiffness of these regions, the model successfully replicated the biomechanical properties of the GBM microenvironment. This approach revealed that stiffer regions enriched in mesenchymal GBM subtypes exhibited greater invasiveness and drug resistance. The study highlighted the potential of 3D bioprinting to create physiologically relevant platforms for investigating the relationship between mechanical properties and tumor cell behavior 108.

Advanced 3D bioprinting method combined with SLA to create a microphysiological system for modeling GBM tumor environments. This system, GlioFlow3D, integrates human brain microvascular endothelial cells and glial cells into hydrogel-based channels within a rigid SLA-printed scaffold 126 (**Figure 4E**). The system is designed to replicate the vasculature and interstitial fluid flow dynamics seen in brain tissue. This model improves traditional methods by avoiding PDMS, which can absorb small molecules like chemotherapeutic agents such as TMZ, affecting the accuracy of drug testing. The 3D-printed system allows for real-time observation of cellular behavior and drug resistance, particularly highlighting the spatial differentiation of tumor cells near vascular structures, which exhibit higher resistance to chemotherapy. By simulating the TME, this method offers a robust platform for investigating complex biology of GBM and drug responses. These models have shown that GSCs near vascular interfaces often exhibit higher resistance to TMZ, mimicking the drug-resistant nature of perivascular GBM cells *in vivo* 126. In another study, a self-organized TME array-on-a-chip was developed by integrating extrusion-based bioprinting with microfluidic technology 138. This platform employs microstructured hydrogel pillars and vascular endothelial cells to form a perfusable vascular barrier around breast cancer spheroids, effectively replicating the TME. The design allows precise regulation of diffusion and shear forces, enabling tumor cell aggregation and self-assembly of vascular networks.

Building upon these advancements, recent studies have further refined bioprinted GBM models by integrating tumor-stromal interactions, vascular mimicry, and GSC enrichment strategies. By employing multi-cellular co-culture systems, researchers have sought to better replicate the TME and enable studies of heterogeneity, invasive behavior, and therapeutic resistance. One such approach involved the coaxial bioprinting of tubular glioma constructs, where glioma cells, U118 were co-cultured with HUVECs in a hydrogel-based fiber-like structure 95 (**Figure 4F**). This model successfully demonstrated that GBM cells actively secrete VEGF and bFGF, leading to the formation of vascular-like networks within the structure. Notably, U118 cells exhibited transdifferentiation into endothelial-like cells, reinforcing the hypothesis that GBM cells themselves contribute to vascular mimicry. However, the lack of fully perfusable vasculature within the bioprinted structure remained a limitation, as effective blood flow is essential for accurately replicating *in vivo* tumor conditions and drug diffusion properties. Similarly, expanding upon these findings, another study investigated the spatial organization of glioma and endothelial cells in a tumor-like construct using coaxial extrusion bioprinting 96. The model integrated a shell-core hydrogel fiber system, with glioma cells in the shell and endothelial cells in the core, allowing researchers to examine tumor-endothelial cell interactions in a structured microenvironment. Results showed that glioma cells within this construct exhibited increased invasion rates and altered gene expression patterns associated with angiogenesis, reinforcing the concept that GBM cells play an active role in shaping their own vascular niche.

In another study, the role of glioma stem cells in tumor progression and therapy resistance was explored through 3D bioprinted glioma cell-laden scaffolds (**Figure 4G**) 94. The model utilized a gelatin-alginate-fibrinogen (GAF) hydrogel scaffold to provide a supportive microenvironment for glioma cells. This environment enabled the maintenance of stemness markers, such as nestin and SOX2, and suppressed differentiation. Compared to traditional 2D cultures, cells within this 3D scaffold demonstrated enhanced epithelial-mesenchymal transition, a key process associated with tumor invasion and recurrence. Importantly, bioprinted glioma cells exhibited significantly higher resistance to TMZ, suggesting that 3D cultures better replicate the therapy-resistant nature of GSCs. Despite these promising findings, the gradual degradation of the hydrogel scaffold posed challenges for long-term experiments, highlighting the need for more stable bioinks that can support prolonged tumor studies. Recently, one notable implementation of this approach introduced a concentric-ring GBM-on-a-chip design, in which patient-derived GBM and endothelial cells were embedded within brain-derived decellularized ECM (BdECM) bioinks. The radial oxygen gradient established across the structure mimicked key tumor-stroma interactions and enabled the reproduction of hallmark pathological features, including hypoxia, pseudopalisading necrosis, and perivascular invasion. Furthermore, the model successfully predicted patient-specific responses to chemoradiotherapy, demonstrating its value as a platform for personalized treatment assessment **(Figure 4H)** 103.

Further efforts to improve GBM modeling have focused on the role of stromal cells in shaping the TME, particularly through bioprinted glioblastoma-mesenchymal stromal cell (MSC) co-cultures 99. This model provided critical insights into how MSCs interact with GBM cells to alter the chemokine landscape, ultimately enhancing tumor invasiveness and immune evasion. Researchers observed that co-cultured glioma cells exhibited increased expression of CCL2, CCL5, and CXCL12, key chemokines involved in tumor progression, recruitment of immune cells, and resistance to therapy. This study emphasized the importance of tumor-stromal interactions in GBM research, yet the static nature of the bioprinted construct limited its ability to replicate dynamic cell migration and fluid exchange observed *in vivo*. Despite these advances, the study highlighted the need for improved methods to integrate functional blood vessels within the bioprinted structure, as tumor models lacking proper perfusion fail to capture key aspects of GBM metabolism and drug response.

The integration of patient-derived tissues into bioprinting-based GBM models requires careful ethical and biosafety evaluation to ensure responsible, safe, and reproducible research practices. Ethically, the use of human-derived materials demands fully informed and voluntary consent, including clear communication about tissue use, downstream applications, long-term storage, associated risks, and potential commercial implications 139. Donor privacy must be strictly protected through de-identification procedures, and equitable representation of diverse patient populations is essential to avoid demographic bias 140. All research involving patient-derived tissues must therefore be conducted under Institutional Review Board (IRB) approval. From a biosafety standpoint, human-derived samples must undergo screening for major pathogens and be handled within appropriate biosafety levels using certified biosafety cabinets, sterile techniques, and standardized operating procedures. Proper sterilization and disposal of biological waste, prevention of cross-contamination during storage, and verification of sample identity through routine cell line authentication are critical to maintaining safety and experimental reliability. For bioprinted constructs intended for translational or clinical applications, additional quality controls such as microbial contamination testing, analysis of residual biomaterials, and evaluation of cell viability and functional performance are required to ensure final product integrity and suitability for downstream use.

### Biomaterials in bioprinted GBM models

The selection of biomaterials is crucial for developing bioprinted models that accurately recapitulate the mechanical and biochemical characteristics of the GBM microenvironment. Hydrogels such as hyaluronic acid (HA) and GelMA are widely utilized due to their brain ECM-mimicking properties and support for cell viability, proliferation, and migration 138, 141. HA is particularly abundant in the brain ECM and plays a key role in facilitating tumor invasion by mediating cell-matrix interactions with glioma cells 199-201. GelMA offers tunable mechanical properties, making it suitable for constructing scaffolds with varying stiffnesses, which is essential for modeling the biomechanical heterogeneity observed in GBM tissue 108, 142.

In recent studies, HA-based hydrogels have been used to encapsulate GSCs, enabling the formation of tumor organoids that closely resemble the *in vivo* characteristics of GBM. These organoids exhibit features such as cellular heterogeneity, stemness, and invasive behavior, providing a powerful tool for studying tumor evolution and drug resistance. The use of GelMA hydrogels in combination with DLP bioprinting has also allowed for the fabrication of multi-stiffness models that replicate the biomechanical heterogeneity of GBM, facilitating the study of stiffness-related tumor behavior 143, 144. To further enhance the fidelity of GBM models, decellularized extracellular matrix (dECM) derived from brain tissue has emerged as a promising biomaterial 103, 145-147. Brain dECM (BdECM) retains a wide range of native ECM components, including collagen IV, laminin, fibronectin, hyaluronic acid, and neurotrophic factors such as BDNF and NGF, which are largely absent in synthetic hydrogels 147. These components provide essential biochemical and structural cues that regulate tumor-stroma interactions, cell migration, and matrix remodeling, facilitating the maintenance of glioma stemness and promoting tumor invasiveness. For example, region-specific dECM from cerebellum and cortex has been shown to differentially regulate astrocyte activation and metabolic rewiring under pathological conditions, suggesting that BdECM can recapitulate not only general but also localized brain microenvironments 145. Moreover, dECM-based hydrogels support enhanced GBM cell viability, long-term culture, and making them valuable for drug screening applications 146. Integration of dECM with biofabrication platforms, such as microfluidic devices or 3D bioprinting, further enables dynamic modeling of glioma progression and matrix-mediated therapy resistance 103. Despite these advantages, the use of BdECM is still limited by batch variability, donor tissue availability, and the lack of standardized decellularization protocols. Nevertheless, it continues to gain recognition as a critical tool for developing more physiologically relevant GBM models.

### Future *in vitro* models

As GBM research progresses, the development of *in vitro* platforms increasingly focuses on incorporating immune system components and systemic organ interactions to better reflect *in vivo* conditions. Two major directions include immune-related organ models and multi-organ systems.

#### Immune-related organ models

Integrating immune components into GBM models offers a promising avenue to study tumor-immune interactions and their impact on disease progression and treatment. Lymph nodes (LNs) and the spleen are particularly relevant due to their central roles in immune surveillance and systemic regulation.

**Figure 5:**
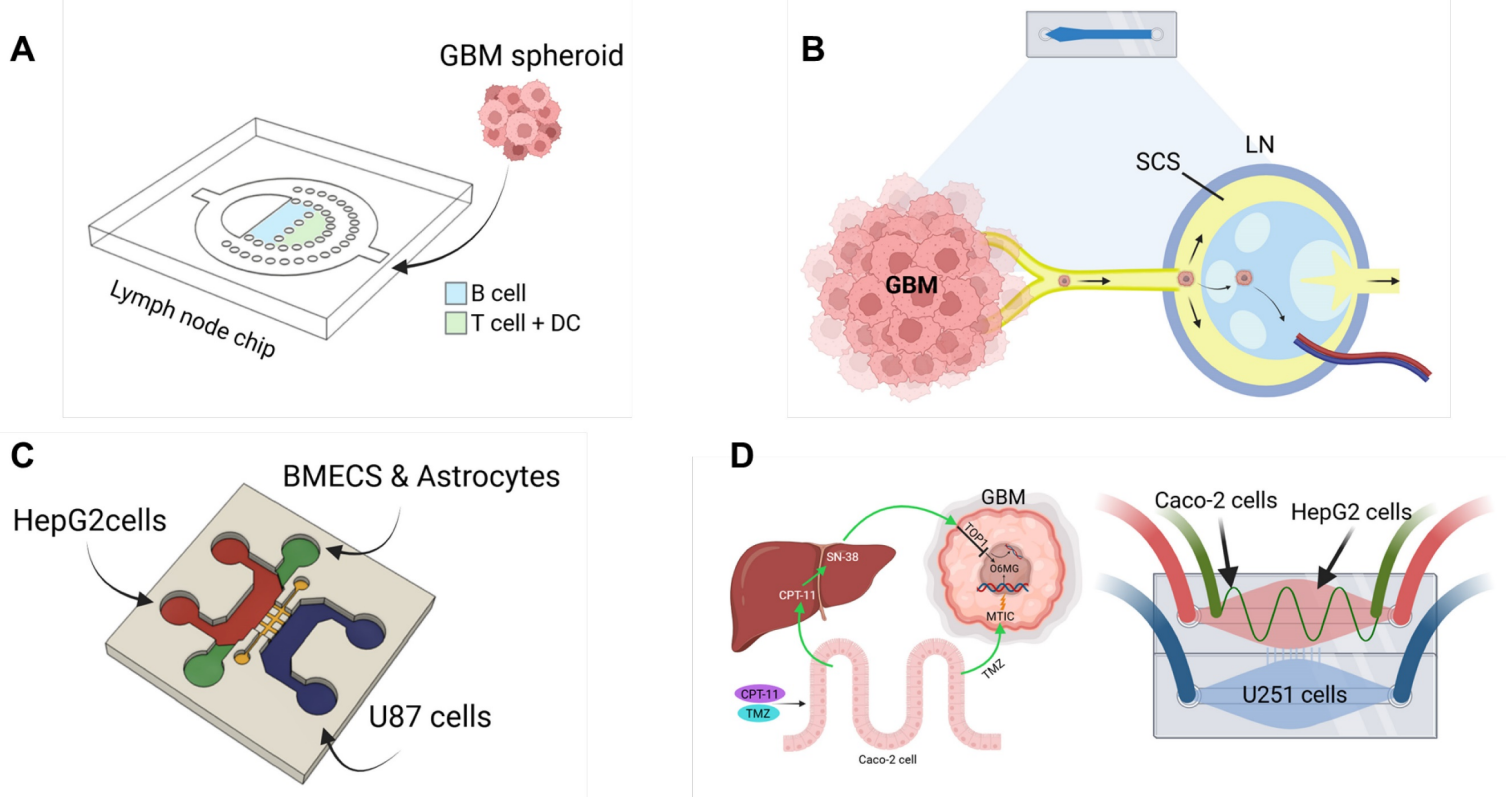
** Future *in vitro* models for GBM research. (A)** Schematics of lymph node-on-a-chip systems. Microfluidic platform mimicking the human lymph node (LN) microenvironment by injecting different immune cell types. Reproduced from under 148 an open-access license, © 2020 Pharmaceutics. **(B)** Tumor- or inflammation-induced lymph node remodeling, characterized by dilation of afferent lymphatic vessels and the subcapsular sinus (SCS). Reproduced from under 149 an open-access license, © 2020 iScience. **(C)** Multi-organ-on-a-chip models incorporating GBM compartments. Schematic and photograph of a multi-interface organ-on-a-chip platform simulating oral drug delivery from the liver (HepG2) through the blood-brain barrier (BMECs and astrocytes) to brain tumors (U87MG), with collagen matrix interconnections. Reproduced with permission from Ref 150 © 2020 Biotechnol. Lett. **(D)** A biomimetic microfluidic system integrating intestinal (Caco-2), hepatic (HepG2), and GBM (U251) compartments for evaluating drug absorption, metabolism, and the synergistic anticancer effects of CPT-11 and TMZ on GBM cells. Reproduced with permission from Ref 88 © 2017 Analyst.

LNs act as biological filters and hubs for immune cell activation, structured into distinct zones for B cells, T cells, and antigen-presenting cells 151, 152. Recent advances in LN-on-a-chip technology replicate these microarchitectures using microfluidic platforms and 3D hydrogels, enabling controlled studies of immune cell dynamics, antigen presentation, and immunotoxicity (**Figure 5A**) 148. Interestingly, the subcapsular sinus-on-a-chip further mimics fluid flow and adhesion cues found in native nodes, offering insights into immune cell trafficking and cancer metastasis (**Figure 5B**) 149. Similarly, the spleen regulates immune responses and red blood cell clearance through its distinct red and white pulp regions 153. The spleen-on-a-chip platform reproduces these filtration and circulatory dynamics using microstructured matrices to evaluate cell deformability, pathogen clearance, and drug effects in hematological disease contexts 154. Despite the technical challenges of preserving immune cell viability and simulating dynamic responses, these immune organ-on-a-chip platforms are expected to greatly enhance the translational relevance of GBM models for immunotherapy testing.

#### Advanced multi-organ systems

To address the complexity of GBM biology, advanced microfluidic platforms are being designed to link the brain tumor model with other functional organ modules. For instance, coupling GBM chips with BBB models enables the study of drug delivery and resistance 155. Additionally, integrating organs such as the liver, which metabolizes drugs, or other immune organs, can help assess the systemic impacts of therapies (**Figure 5C**) 150. One integrated microfluidic platform simulating intestine-liver-GBM interactions demonstrated that dual-drug combinations, such as irinotecan and TMZ, offer superior efficacy by accounting for metabolic and drug delivery (**Figure 5D**) 88. Similarly, a liver-brain-GBM chip showed how hepatic metabolism and BBB permeability modulate drug responses to agents like paclitaxel, capecitabine, and TMZ 150. These multi-organ platforms offer a system-level approach to studying GBM, bridging the gap between conventional *in vitro* assays and the complexity of human physiology. They are expected to enhance predictive accuracy for therapeutic screening and accelerate the development of personalized treatments 156.

## Microfluidic GBM model for drug screening

### Temozolomide (TMZ) as a single agent

TMZ is the primary chemotherapeutic agent used in the standard-of-care regimen for GBM, typically administered after surgical resection and radiotherapy 67. TMZ exerts its cytotoxic effect by methylating guanine residues in DNA, leading to replication errors and cell death 161. However, therapeutic resistance significantly limits its efficacy. This resistance primarily arises from the DNA repair enzyme MGMT, which reverses TMZ-induced DNA damage, along with the activation of alternative DNA repair mechanisms 162.

**Figure 6:**
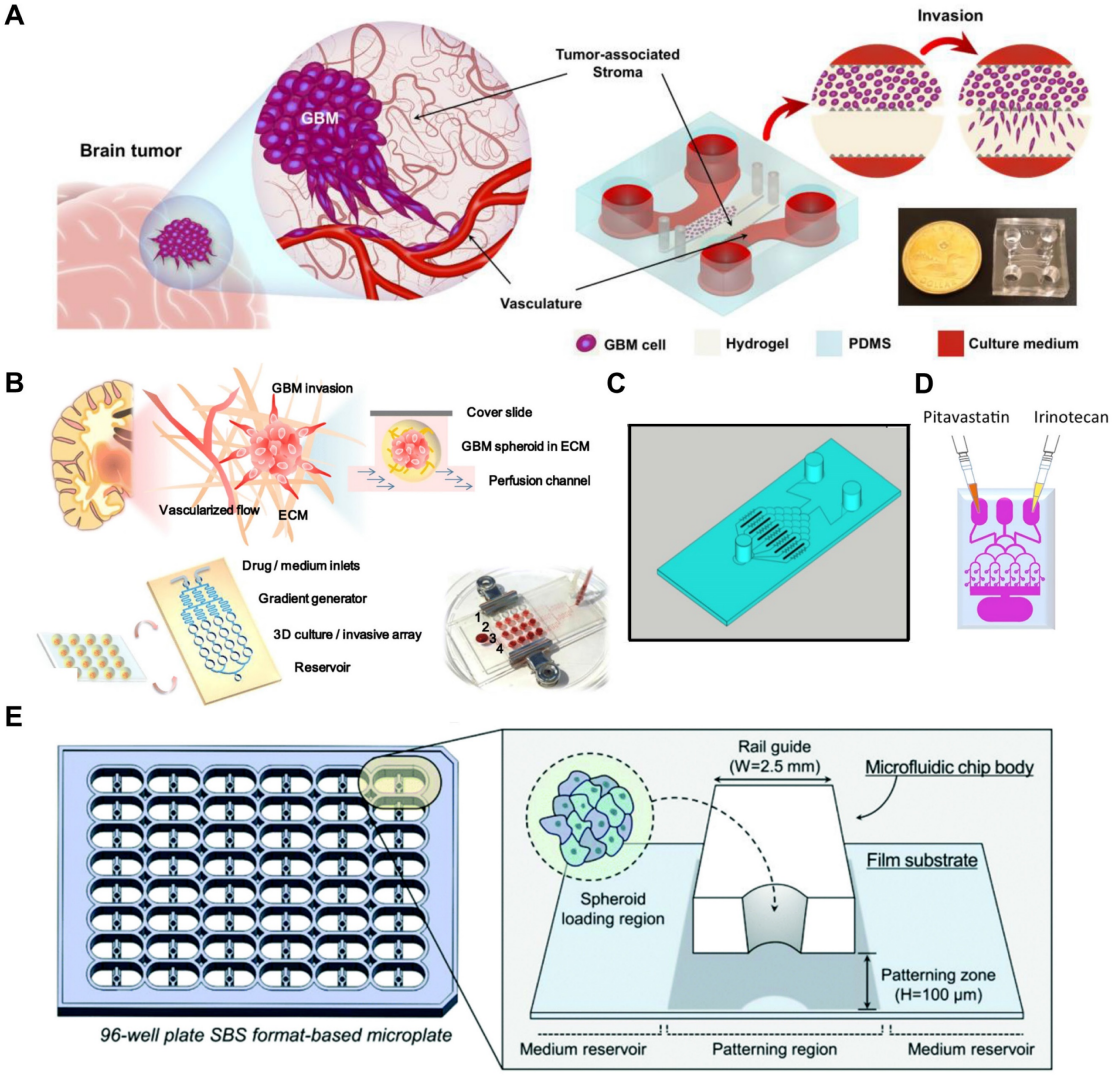
** Microfluidic-based platforms for evaluating GBM drug response and combination therapies. (A)** Schematic of GBM and the tumor-on-a-chip model featuring microfluidic chips with tumor and stromal compartments integrated with drug delivery channels. Reproduced from under 157 an open-access license, © 2020 Int. J. Mol. Sci. **(B)** Schematic of a 3D perfusion microfluidic chip for the evaluation of GBM cell invasion and drug response. Reproduced with permission from Ref 158. © 2018 Biomed. Microdevices. **(C)** Gradient microfluidic chip featuring dual inlets and microwell arrays for TMZ and BEV screening via drug concentration gradients. Reproduced from under 159 an open-access license, © 2018 Sci. Rep. **(D)** DPEGDA-based brain cancer chip featuring gradient channels and 24 microwells for high-throughput drug combination analysis. Reproduced from under Ref 86 an open-access license, © 2016 Sci. Rep. **(E)** Schematic of a standardized microfluidic platform for modeling GBM spheroid-induced angiogenesis and drug screening. Reproduced with permission from Ref 160 © 2019 Lab Chip.

To address these challenges, advanced preclinical models have been developed to better evaluate TMZ efficacy. Microfluidic platforms allow real-time analysis of drug-induced cellular responses, such as changes in adhesion at the single-cell level, providing insights beyond traditional viability assays 163. In one study, U87MG and U251 GBM cell lines were embedded within a microfluidic chip to assess the combined effects of TMZ and simvastatin. Drug efficacy was quantified via viability assays, immunofluorescence staining for apoptosis, and analysis of cell invasiveness, enabling simultaneous investigation of invasion, apoptosis, and autophagy pathways 157 (**Figure 6A**).

Other microfluidic systems replicate key aspects of the TME, including spatial tissue organization and continuous perfusion. These platforms support parallel manipulation of tumor spheroids and enable assessment of cell proliferation, viability, and invasiveness under dynamic drug exposure. Such models have demonstrated the effectiveness of agents like resveratrol and TMZ in reducing GBM invasiveness, underscoring their utility for personalized medicine and therapeutic screening (**Figure 6B**) 158. Furthermore, a 3D bioprinted GBM vascular model was developed to better capture tumor heterogeneity and resistance patterns. This platform allows long-term culture and has shown that TMZ initially induces tumor regression, followed by recurrence, a phenomenon poorly modeled in 2D or suspended spheroid systems. By more accurately replicating *in vivo*-like tumor behavior, the bioprinted model provides a robust platform for optimizing TMZ-based therapies in a personalized context 100.

### Combination therapy

Combination therapy has emerged as a promising strategy to overcome the limitations of monotherapy in GBM treatment. In particular, co-administration of TMZ with immune checkpoint inhibitors (ICIs) targeting PD-1 or CTLA-4 has demonstrated encouraging results in preclinical and early clinical studies. These regimens aim to restore anti-tumor immune surveillance while counteracting the immunosuppressive TME characteristic of GBM 164.

Another widely studied combination involves TMZ and bevacizumab, a monoclonal antibody targeting VEGF. Bevacizumab disrupts angiogenesis and enhances progression-free survival by impairing tumor vascular integrity. Microfluidic models have been instrumental in evaluating such combinations by enabling physiologically relevant drug exposure conditions. For example, a microfluidic platform incorporating 3D patient-derived GBM spheroids enabled precise control of drug diffusion and independent channel testing. This setup allowed detailed analysis of TMZ and bevacizumab interactions and their effects on tumor cell proliferation and viability (**Figure 6C**) 159.

Moreover, high-throughput drug screening is facilitated by microfluidic devices that allow parallel testing of multiple agents. In one study, a PEGDA-based hydrogel system supported the formation of uniform 3D GBM spheroids and was used to screen various drug combinations. The pairing of pitavastatin with irinotecan showed enhanced anti-tumor efficacy, demonstrating the potential of the platform in personalized medicine and rapid drug discovery (**Figure 6D**) 86.

In another example, a microfluidic chip incorporating pneumatic microstructures was used to trap U251MG GBM cells and generate uniform tumor arrays within a collagen matrix. This device enabled long-term culture for over a month and supported repeated drug testing cycles. Chemotherapeutic agents such as vincristine and bleomycin were evaluated using assays for viability and mitochondrial membrane potential, offering insights into drug-induced apoptosis 165. These integrated systems collectively provide versatile platforms for investigating synergistic drug effects, understanding tumor heterogeneity, and developing optimized combination regimens tailored to individual patient responses.

Recently, the Sphero-IMPACT platform has been used to evaluate combination therapies in GBM, including anti-angiogenic drugs. In a co-culture model of U87MG spheroids and HUVECs within a fibrin matrix, endothelial invasion was observed and effectively inhibited by bevacizumab and sunitinib. This highlights the utility of platform in modeling tumor angiogenesis and screening vascular-targeted therapies in a reproducible, high-throughput format (**Figure 6E**) 160.

### Immunotherapeutic investigations

Immunotherapy has emerged as a central focus in GBM treatment strategies due to the poor prognosis of disease and resistance to conventional therapies 166. The GBM microenvironment shaped by the interplay of immune cells such as NK cells, TAMs, and T cells, plays a critical role in modulating immune responses and therapeutic efficacy 19. However, the presence of the BBB significantly limits the delivery and efficacy of immunotherapeutic agents 167.

Microfluidic models have enabled the recreation of key features of the TME—such as immune cell infiltration, hypoxia, and cell-cell interactions—under physiologically relevant conditions. In one study, patient-derived GBM cells were used to develop an *ex vivo* microfluidic platform for personalized evaluation of responses to immune checkpoint inhibitors (ICIs), particularly anti-PD-1 therapies. This system revealed that mesenchymal GBM subtypes exhibited increased CD163⁺ TAMs and PD-1/PD-L1 signaling, contributing to immune evasion. Dual inhibition using nivolumab and a CSF-1R inhibitor (BLZ945) reduced TAM density and restored CD8⁺ T-cell activity, offering a tailored immunotherapeutic strategy 91. Angiogenesis-focused microfluidic models have also elucidated immune-vascular crosstalk in GBM. A 3D angiogenesis-on-a-chip system demonstrated that TGF-β1 and αvβ3 integrin-mediated signaling drive macrophage polarization towards an M2 phenotype, promoting inflammation-induced angiogenesis. Targeting this pathway with combined αvβ3 and TGFβ-R1 inhibition suppressed neovascularization and modulated immunosuppressive signaling 168.

Advances in CAR-T cell therapies have further benefited from microfluidic technologies. BBB and blood-brain-tumor barrier (BBTB)-on-chip models derived from iPSCs have been used to test CAR-T constructs targeting EGFRvIII-overexpressing GBM cells. These platforms enabled analysis of CAR-T cell cytotoxicity, extravasation, and barrier integrity, demonstrating distinct behaviors of CAR-F263 and CAR-F269 variants 169, 170.

Additionally, antibody-drug conjugates (ADCs), which deliver cytotoxic payloads via tumor-specific monoclonal antibodies, have been evaluated using microfluidic models. Combining ADCs or CAR-T cells with ICIs on these platforms provides a unique opportunity to investigate resistance mechanisms and optimize combination regimens. Despite their utility, microfluidic models for GBM immunotherapy remain underdeveloped compared to those for other cancers. Challenges include replicating the full complexity of the immune microenvironment and the BBB. Limitations such as artificial materials, lack of immune diversity, and non-physiological cell ratios often reduce translational relevance. To advance GBM immunotherapy, there is a pressing need for standardized, reproducible microfluidic systems that faithfully recapitulate the immune and vascular microenvironments. Such platforms will be critical for evaluating new immunotherapeutic combinations and accelerating clinical translation.

While these examples highlight the potential of microfluidic platforms to inform immunotherapy design, current systems still face significant limitations in faithfully modeling immune-tumor dynamics 171. Despite recent advances, incorporating immune cell populations such as TAMs and T cells into GBM-on-a-chip platforms remains a significant challenge. Primary immune cells offer physiological relevance but are limited by variability and poor long-term stability. On the other hand, immortalized cell lines provide consistency yet fail to capture the dynamic, context-dependent behavior of native immune cells. As a result, many platforms lack the cellular diversity and functional complexity necessary to reflect authentic immune-tumor interactions. Moreover, immune integration is often constrained by oversimplified co-culture systems, non-physiological ECM materials, and the absence of critical structural components such as the BBB and tumor vasculature 172. These elements are essential for mediating immune infiltration, polarization, and therapeutic response but are frequently underrepresented or excluded altogether. Overcoming these limitations will be vital for enhancing the biological fidelity of GBM-on-chip systems and enabling their application in immunotherapy testing.

### Phototherapy

Phototherapy is an advanced cancer treatment that utilizes light to specifically target and kill tumor cells. It operates through two main methods: photodynamic therapy (PDT) and photothermal therapy (PTT). PDT involves the use of a photosensitizer, a light-sensitive compound that produces ROS upon activation by specific wavelengths of light. These ROS cause localized damage to tumor cells, ultimately leading to cell death 173, 174. PTT, in contrast, relies on photothermal agents to absorb light energy and convert it into heat. This localized heating raises the temperature in the TME, resulting in the destruction of cancer cells through protein denaturation and cell membrane disruption 175. While PDT requires the combined action of light, oxygen, and a photosensitizer, PTT can often function independently of additional agents, though the inclusion of photothermal agents can significantly enhance its efficacy 176.

PDT has been effectively employed to treat various conditions, including skin disorders, ocular diseases, and cancers. Recent advancements have focused on improving its precision and therapeutic outcomes through nanotechnology. By integrating photosensitizers with nanomaterials, researchers have been able to enhance the generation of ROS and improve the targeted delivery of PDT agents. For example, microfluidic systems—miniaturized devices that allow precise control of experimental conditions—have been used to study the effects of PDT on GBM cells. One study utilized a microfluidic chip to explore how varying levels of photosensitizers, oxygen, and light influence therapeutic outcomes. Results indicated that increasing these factors significantly enhanced the cytotoxic effects of PDT. Another study demonstrated the potential of methylene blue (MB)-conjugated polyacrylamide nanoparticles (PAA NPs)-functionalized nanoparticles, optimized with a polyethylene glycol dimethacrylate (PEGDMA) cross-linker to enhance ROS production for PDT. The design minimized self-quenching, improved oxygen permeability, and was evaluated using advanced analytical tools to determine ROS composition and efficacy in a microfluidic platform. This approach identified near-optimal MB loading for effective light-dose-dependent killing of C6 GBM cells, highlighting the promise of combining nanotechnology with PDT 177.

Microfluidic technologies have become invaluable in phototherapy research, as they allow scientists to replicate complex tumor environments and evaluate therapeutic strategies in controlled settings. For instance, hydrogel-based microfluidic systems were utilized to develop a co-culture device for studying PTT and cancer cell migration, employing 10 w/v% GelMA hydrogels as semi-permeable barriers for culturing MCF7 breast carcinoma and U87MG GBM cells. This system demonstrated gold nanorod-mediated PTT effects on GBM and showed significant migration of U87MG cells toward VEGF-treated GelMA microchannels. Such hydrogel microfluidic devices provide a robust platform for evaluating PTT efficacy and analyzing migration behaviors of cancer cells with different motilities 178. While PDT and PTT have advanced cancer phototherapy, their dependence on light restricts treatment of deep brain tumors. Recently, piezoelectric dynamic therapy (PZDT) has emerged as a non-optical, ultrasound-activated therapeutic strategy. Upon exposure to ultrasound, piezoelectric materials generate localized electric fields and induce charge separation, which in turn triggers a cascade of biochemical reactions, including enhanced ROS generation and disruption of cellular electrochemical homeostasis. Importantly, ultrasound exhibits superior tissue penetration compared to light, enabling PZDT to exert therapeutic effects in deep tumor regions that are largely inaccessible to PDT and PTT 179.

## *In vivo* model for GBM treatment

**Figure 7:**
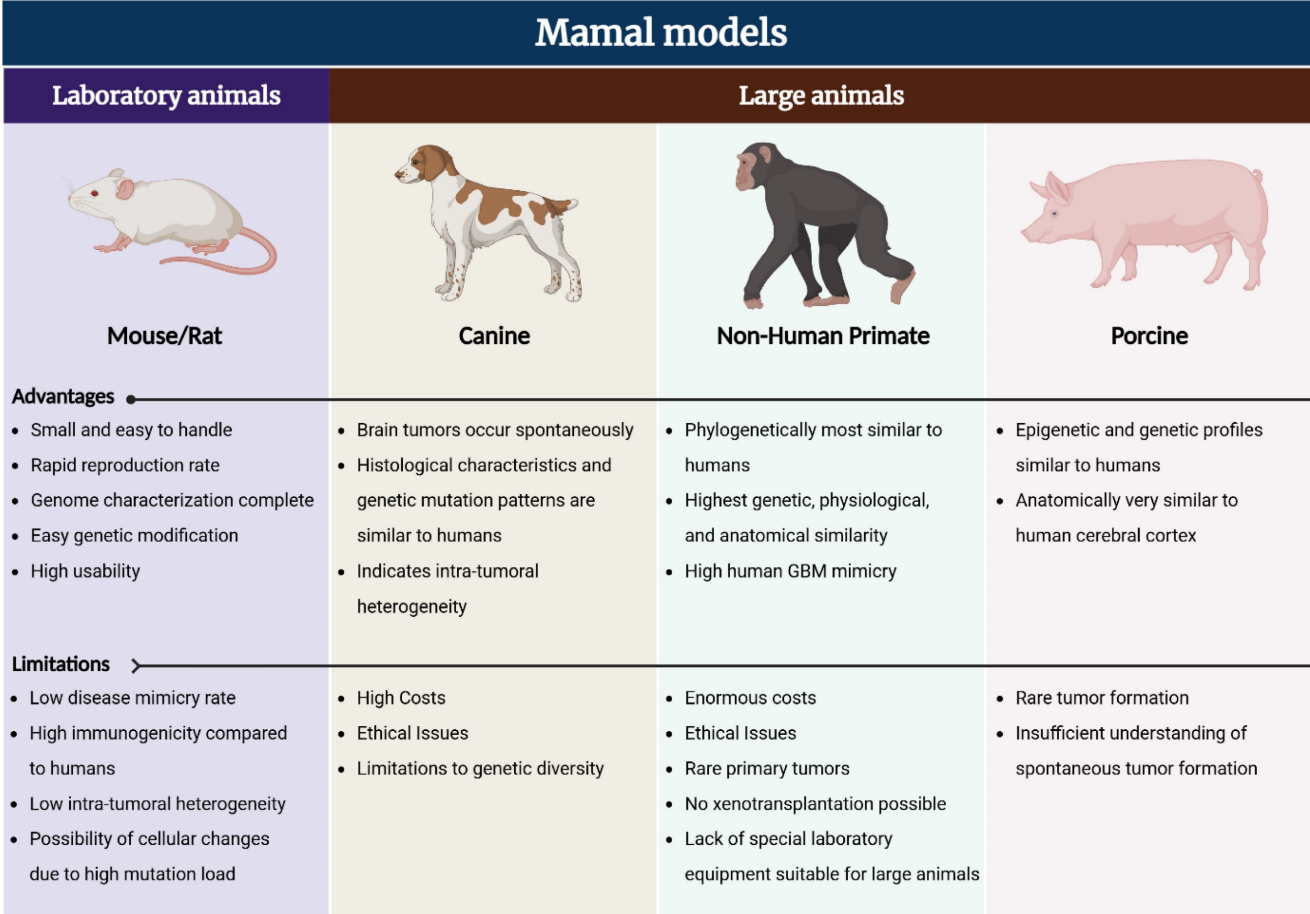
Comparative overview of mammalian animal models used in GBM research

The purpose of the preclinical process is to select final drug candidates to be applied to clinical trials through *in vitro* and *in vivo* studies and to determine safe doses for phase 1 clinical trials. Drug candidates screened through *in vitro* studies proceed to *in vivo* evaluations, where anticancer activity is verified and drug toxicity and pharmacokinetics studies are conducted 180. *In vivo* models can replicate tumor behavior by reflecting actual diseases, which helps verify the efficacy and safety of drugs and discover new therapeutic strategies. Particularly, models that emulate key carcinogenic features, including the biological microenvironment, immune and inflammatory responses, and angiogenesis, provide new perspectives on tumor biology and facilitate a better understanding 181, 182. This section provides a comprehensive review of the diverse *in vivo* animal models used in GBM research **(Figure 7)**.

### Mouse models

**Figure 8:**
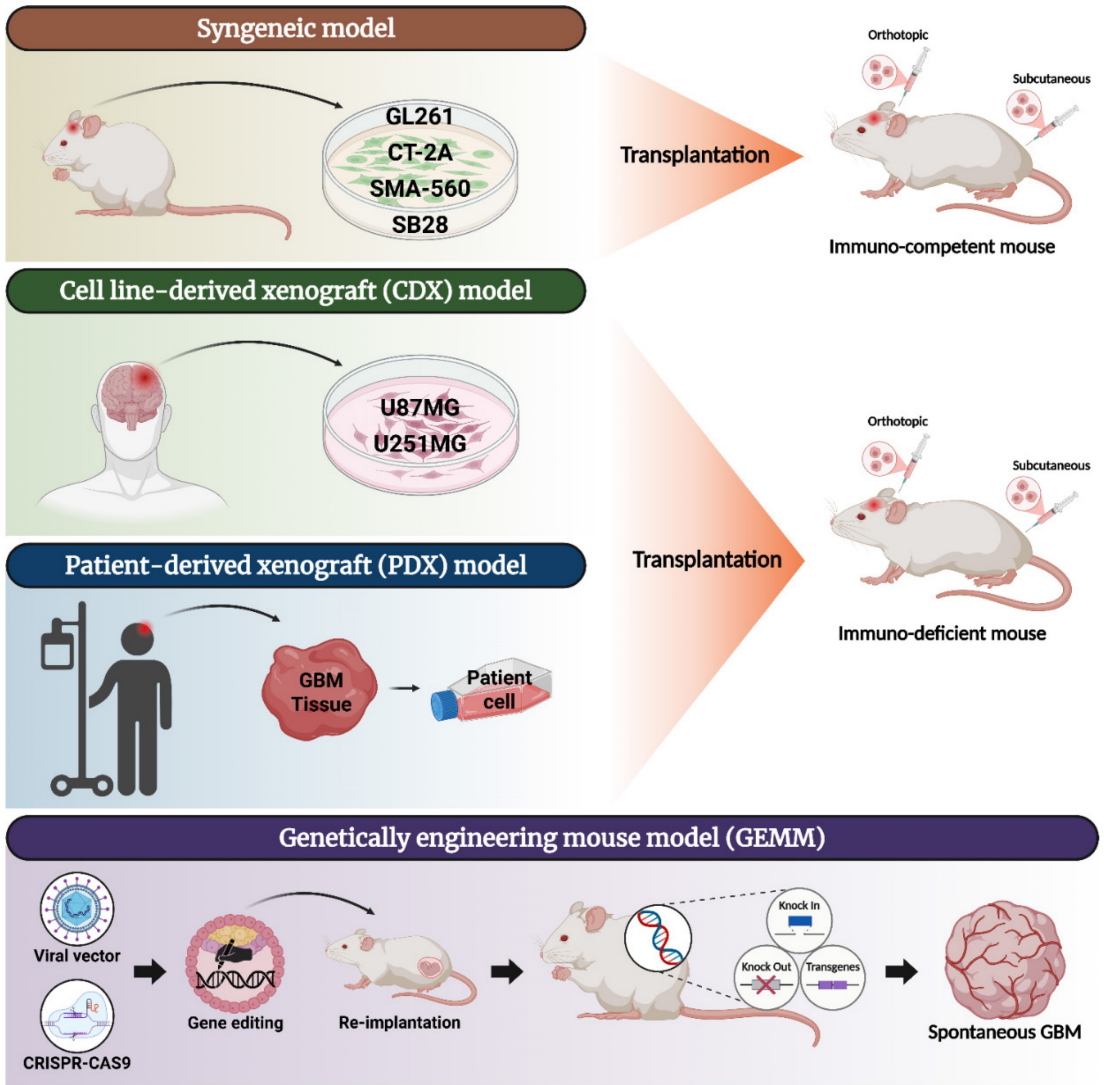
Classification of *in vivo* GBM mouse models based on tumor induction method.

The mouse model is the most commonly used *in vivo* experimental model due to its relatively small size, rapid breeding essential for sustained experimental maintenance, and the thorough characterization of the mouse genome which provides experimental convenience 181. Mice have been used in the development of several therapeutic strategies for human GBM and have provided valuable data, as they can mimic the complex genetic and physiological aspects of cancer and generate neoplastic tumors while maintaining tumor-host interactions. Particularly, mouse models are amenable to genetic manipulation in various ways, making them useful in assessing the effects of different mutations on tumorigenesis and treatment responses **(Figure 8)** 183, 184.

#### Syngeneic models

Syngeneic models are the oldest and most widely used preclinical models, being homogeneous *in vivo* models used between genetically identical individuals. This model is particularly useful for studying the interaction between the TME and the host immune system because it is immunocompetent, unlike other *in vivo* models that lack the immune capacity of experimental animals 185. In the syngeneic GBM model, murine-derived brain tumor cell lines derived by various methods are used, such as GL261, CT-2A, SMA-560, and SB28.

##### GL261

GL261 is histologically very similar to human GBM, exhibiting features such as anaplastic and pleomorphic cells, active mitosis, and atypical nuclei, and as a model with a long history, tumor progression stages have been well studied, making it easy to interpret results and predict potential therapeutic responses 186, 187. GL261 has been shown to be resistant to TMZ *in vitro* and expresses stem cell markers including mesenchymal properties such as CD133, CD44, nestin, and SOX2 *in vivo* 188, 189. This allows it to be used to study the development of human GBM and to study glioma stem cells involved in chemotherapy and radiotherapy resistance.

The GL261 mouse model has been used in studies to discover novel therapeutics to develop standard treatments that do not significantly improve the overall survival rate of GBM patients 190, 191 and to develop administration methods to increase the delivery efficiency of TMZ 192. It has also been actively utilized in immunotherapy development studies such as checkpoint-related immunotherapy and combination therapy with TMZ 193, 194, evaluation of the association between TMZ treatment and immune cell phenotypic changes in the TME 195, and alleviation of GBM-induced immunosuppression 196. In addition, it has been widely used in studies to evaluate anti-GBM effects based on vaccines 197-199.

##### CT-2A

CT-2A, like GL261, recapitulates the characteristics of high-grade gliomas, including high cell density, pseudo-palisading necrosis, rapid abnormal mitosis, and microvascular proliferation, and exhibits proliferative and invasive properties similar to human GBM 200, 201. The most notable feature of CT-2A is that given growth conditions for brain tumor stem cells, stemness can be enhanced, leading to significantly increased proliferation and invasiveness. It expresses stem cell markers such as CD133, Oct4, and nestin, which may be useful for studying glioma stem cells 201. Additionally, the CT-2A glioma model is more resistant to checkpoint blockade therapy than the GL261 model 202, 203.

Due to their similar properties to GSCs, the CT-2A model is often utilized for tumor stem cell research in an immune-competent environment. The NS/CT-2A model, developed based on CT-2A cells cultured in neurospheres rather than monolayers, exhibited more abundant GSC markers, increased angiogenesis, and shorter mouse survival. It also showed an aggressive phenotype with decreased CD8+ T cells and increased regulatory T cells, which enhanced immunosuppressive properties, suggesting a clinically relevant platform, similar to the low immunogenicity of human GBM 204. In addition, CT-2A has been actively utilized in various anti-GBM immunotherapy studies, including validating the combined effect of immunotoxin D2C7-IT and αCTLA-4/αPD-1/αPD-L1 therapy in a CT-2A mouse model overexpressing mutant EGFRvIII, a well-known glioma antigen 205, evaluating the efficacy of genetically engineered T cells targeting EGFRvIII in glioma stem cells 206, and evaluating the antitumor activity of mRNA-based multifunctional CAR T cells against malignant brain tumors 207.

##### SMA-560

SMA-560 histologically resembles dysplastic astrocytoma with highly cellular features showing focal necrosis and sporadic vascular proliferation. However, since nuclear atypia and strong expression of glial fibrillary acidic protein (GFAP) are also present, it is considered to morphologically reproduce human glioma 208, 209. SMA-560 is resistant to TMZ both *in vitro* and *in vivo* and is slightly more sensitive to radiotherapy 210. It also expresses the immunosuppressive molecule TGF-β, similar to human GBM, allowing the study of immunosuppressive effects on immunotherapy 211.

The SMA-560 model, characterized by TGF-β expression, has been used to investigate the correlation between the TGF-β pathway involved in angiogenesis, invasiveness, and immunosuppression and VEGF inhibition 212. Additionally, this strategy has been applied to inhibit TGFβ using two antisense oligonucleotides targeting TGFβ1 and TGFβ2 213. In addition, there are studies that have evaluated tumor growth rates and toxicity by modifying SMA-560 cells. Genetic modification of tumor cells to secrete IL-2, IL-4, and TNFα enhanced antitumor immune responses and improved mouse survival 208. Likewise, expression of a soluble CD70 ligand that stimulates CD27 signaling increased CD8⁺ T cell infiltration and extended survival 214. It has also been used to study the reversal of the immunosuppressive GBM microenvironment via local delivery of IL-12 215.

##### SB28

SB28 histologically shows hypervascularization and high cellularity, and infiltrates into the surrounding normal brain parenchyma 216. Compared to other syngeneic mouse models such as GL261, SB28 is a more accurate representation of human GBM due to its lower immunogenicity, characterized by reduced tumor-infiltrating T cells, diminished MHC I expression, and significantly lower mutational load, making it an attractive model for the development of potential immunotherapeutic agents in preclinical trials 216-218.

The SB28 mouse model has been frequently used in immunotherapy and radiotherapy development studies. When radiation therapy was combined with a TGF-β inhibitor or anti-PD-L1 therapy, sensitivity was increased and survival was prolonged 219, and combination therapy with anti-CD40 monoclonal antibody and cyclooxygenase-2 (COX-2) inhibitor celecoxib was confirmed to increase anti-glioma activity by promoting type-1 immunity in both myeloid cells and T cells 218. In addition, the effects of combination of mitogenic targeting drugs (spindle assembly checkpoint activators) and immune checkpoint blockade therapy on the TME and immunotherapy sensitivity were evaluated 220, and studies were also conducted on the association between immune evasion, a characteristic of GBM, and the intrinsic characteristics of the tumor and the special immune context of the brain using immune checkpoint inhibitors (ICIs) and the possibility of overcoming them 221, 222.

Although several syngeneic mouse models have been developed for the mechanism and successful preclinical studies of human GBM, they have limitations. The most significant limitation is that mice cannot guarantee that they accurately mimic real human diseases due to important genetic, immunological, molecular, and cellular differences from humans 223, 224. In particular, most mouse-derived cell lines, such as GL261, CT-2A, and SMA-560, exhibit high immunogenicity compared to the low immunogenicity of human GBM 203, 208, 217, 225. This property may not be suitable for studies for immunological evaluation because it increases sensitivity to immunotherapeutic agents. Unlike human GBM, which has a relatively low mutational load compared to other aggressive malignancies, GL261 has a high tumor mutational load with over 4000 non-synonymous exome mutations and a large number of predicted neoepitopes 217, 226. It is therefore vulnerable to cellular changes that may occur during long-term cell culture *in vitro* 227. In addition, CT-2A tends to have low invasion into the surrounding brain parenchyma, unlike human GBM, and SB28 is a homogenous cell line, in contrast to the high intra-tumoral heterogeneity of human GBM 225, 228. As these significant differences from the properties and microenvironment of actual human brain tumors contribute to the failure of human clinical trials, development of advanced preclinical models that can most closely mimic human GBM is essential.

#### Xenograft models

Xenograft models have been developed to more closely reproduce human diseases and have long been used to study tumor development by subcutaneously or intracranially transplanting human-derived tumor cells into immunodeficient mice 229. Since the biggest challenge in transplanting cells or tissues from other species is xenogenic immune rejection, immunodeficient mice are mainly used as hosts. Although immunodeficient mice are diverse, they are broadly classified into three types based on immunological differences: 1) athymic nude mice that do not produce T cells; 2) non-obese diabetic severe combined immunodeficiency mice (SCID and NOD-SCID) with depleted T and B cells; 3) NOD-SCID IL2R-γ null mice (NOG and NSG) with depleted T and B cells and extremely low NK cell activity 230. Xenografts include cell line-derived xenografts (CDX) that transplant human GBM cell lines and patient-derived xenografts (PDX) that transplant patient-derived cells. Human GBM cell lines, including U87MG and U251MG, which are widely used in CDXs, are easy to handle and can mimic the histopathology of human GBM, and PDXs have improved ability to more closely reproduce actual disease, making xenograft models more likely to reproduce human GBM than syngeneic models 225.

##### Cell line-derived xenograft models

##### U87MG

The U87MG cell line is one of the most widely used human GBM cell lines and is thought to have genetic similarity to human GBM. It has mutations in hTERT, ATRX, and PTEN, no mutations in p53 and IDH1, and a methylated MGMT status, which are the same characteristics as human GBM 231, 232. In addition, U87MG expresses CD133, forms neurospheres, and can be used in glioma stem cell research 233.

Since its establishment, the U87MG cell line has been widely used for a long time, and the xenograft mouse model has also been used in various research fields. Studies have been conducted to elucidate the effects and mechanisms of natural product extracts on glioma 234, 235, or to develop therapeutic strategies for human GBM by enhancing the delivery ability of anticancer drugs such as cisplatin or doxorubicin in combination with serum albumin 236, 237. Additionally, it has been used in a wide range of studies, including comparative studies of bevacizumab doses for the treatment of gliomas 238, and molecular response studies using bevacizumab-resistance models 239.

##### U251MG

The U251MG cell line has a high proliferative capacity, most cells stain with Ki-67, and like U87MG, express CD133 and can form neurospheres 233, 240. Genetically, it has mutations in hTERT, PTEN, and p53, but no IDH1 mutation and a methylated MGMT status 232. It has the advantage of reproducing the histopathology of human GBM, such as invasive infiltration, cellular atypia and mitotic figures, and palisading necrosis 240, 241.

U251MG has also been used in studies to improve standard treatments, such as intracranial direct administration of bevacizumab 242 and combined effects of metformin and TMZ 243, as well as various functional studies to induce apoptosis of U251MG cells through modulation of proteins such as NF-E2-related factor 2 (Nrf2) 244 and hypoxia-induced BAX 245. In addition, a comparative study on toxicity and anticancer efficacy between liposomal doxorubicin and free doxorubicin via convection-enhanced delivery (CED) was also conducted 246.

However, there are quite a few problems with xenograft models using cell lines. The first problem that arises is that they are limited in evaluating immunotherapy because they must be modeled in immunodeficient mice 225. In addition, human GBM-derived cell lines consist of relatively homogeneous populations and thus lack the intratumor heterogeneity observed in patient tumors. Furthermore, long-term culture in serum-containing media can lead to genetic drift and transcriptomic alterations, limiting their ability to accurately recapitulate human GBM 247.

In particular, the U87MG model has many shortcomings. U87MG tumors transplanted into mice are well-defined as they are surrounded by reactive stellate cells without diffuse infiltration 2, 240, 241. This is a significant difference from human GBM, which is characterized by diffuse infiltration. Additionally, necrotic features are rare, and there is little pseudo-palisading patterns or immune cell infiltrations 229. Relative to human GBM, the model demonstrates increased vascularization, which may enhance therapeutic delivery. In contrast to clinical GBM, it remains responsive to both radiation and temozolomide treatment 248-250. Another drawback of the U87MG cell line is the controversy over its authenticity. This issue was first raised when the laboratory that first isolated the U87MG cell line discovered that the U87MG cell line from the American Type Culture Collection (ATCC) had a different DNA profile from the original cells 77. Therefore, although the U87MG cell line has been widely used and has contributed greatly to neuro-oncology research, concerns about authenticity and reproducibility have diminished its preference.

The U251MG cell line also has its drawbacks. Like U87MG, it is known to respond to both TMZ and radiation treatment 251-253. The biggest problem is that in 1999, it was reported that the U373MG cell line was cross-contaminated with the U251MG cell line 241. This is a similar issue to the authenticity of the U87MG cell line. Moreover, when the U251MG cell line was cultured for a long time, genetic changes accumulated, causing changes in various phenotypes, including cell morphology, proliferation rate, and cell surface marker expression 254. Therefore, studies using the U87MG and U251MG cell lines may require careful approach and cell line validation.

##### Patient-derived xenograft models

The patient-derived xenograft (PDX) mouse model is one in which tumor tissue is obtained from patients, processed into single-cell suspensions or pieces, and injected immediately into the subcutaneous or intracranial microenvironment of mice 255. PDXs are also human-derived cells, so immunodeficient mice must be used to prevent tumor rejection.

The main advantage of PDX models is that they can faithfully reproduce the characteristics of actual disease. Studies using GBM PDX models have shown that they reproduce the characteristics of human GBM, such as diffuse invasion, pseudo-palisading necrosis, and endovascular proliferation 36, 256. Additionally, the characteristics and growth patterns of each GBM subtype were identified. PDX models using mesenchymal (MES) cells had a higher proliferation rate and increased invasiveness and vascularity compared to models using proneural cells 256-259. This implies that the PDX model reflects the physiology of human GBM.

PDX models may have problems reproducing disease depending on the implantation location, so this must be carefully considered when designing. Orthotopic intracerebral implantation is preferred because it preserves the physiological environment of the BBB and cerebrospinal fluid, whereas subcutaneous implantation often fails to support normal tumor growth due to the markedly different microenvironment outside the brain 260. A study investigating the genetic, epigenetic, and histopathological stability of orthotopic intracranial transplantation GBM PDX consistently reproduced primary tumor characteristics in contrast to subcutaneous transplantation, and long-term stability was demonstrated without evidence of mouse-specific tumor evolution 261. Therefore, orthotopic PDX models can be useful for studies that measure and predict responses to novel therapies.

PDX models have been widely used in various studies due to their high reliability in reproducing human GBM. This model has been used to evaluate the anti-GBM effects and mechanisms of various potential agents, including diallyl trisulfide (DATS), a histone deacetylase inhibitor with reported anti-tumor activity 262, lisavanbulin alone or in combination with RT/TMZ 263, and combination therapy with a GSK-3 inhibitor and 1-[2-chloroethyl]-3-cyclohexyl-1-nitrosourea (CCNU) for chemo-resistant GBM 264. Furthermore, studies such as the development and characterization of the GBM orthotopic PDX mouse model panel for drug efficacy evaluation have been conducted 265. As such, the PDX model has been used in a wide variety of studies, helping to derive valuable results.

However, the PDX model also has disadvantages. First of all, as with CDX, because it uses immunodeficient mice, studies on the immune system and immune response to treatment cannot be conducted 225. In addition, the establishment of patient-derived cells that can be used for xenografting is not easy and can take a long time, and the establishment success rate varies depending on the method and experience of each laboratory 266-268. One of the problems encountered while culturing patient-derived cells is that GSC subpopulations within GBM can disappear after prolonged exposure to serum. GSCs differentiate in serum-containing conditions and can lose many primary tumor characteristics, which is a critical problem because GSCs are important for maintaining intra-tumoral heterogeneity, a hallmark feature of GBM 247. Additionally, the use of patient-derived cells can be a double-edged sword. While PDX models can facilitate individualized studies because they use cells from a single individual patient, there can be significant variability between individual models, limiting standardization and reducing reproducibility of experimental results 184, 268.

#### Genetically engineered mouse models

As research on GBM continues, several genes such as EGFR, PTEN, and BRAF, which are commonly mutated, have been discovered, which are correlated with poor prognosis of patients 269-271. Based on the association between these genetic changes and tumor progression, genetic research has become active and has led to the development of genetically engineered mouse models (GEMMs) 272. GEMMs can be created by altering the genome and generating tumors through a variety of methods, including knockout and conditional knockout, replication-competent ALV LTR with a splice acceptor (RCAS), clustered regularly interspaced short palindromic repeats (CRISPR)-Cas9 technology, and other viral technologies 273. Among these, CRISPR/Cas9 technology has significantly contributed to the creation of diverse GEMMs by enabling manipulation of single and multiple gene mutations 274.

GBM GEMMs have distinct advantages over other *in vivo* mouse models. Since these models induce tumors through genetic modification rather than direct injection of tumor cells into the brain, tumor development occurs in a manner similar to human GBM, allowing for a more accurate recapitulation. In addition, since there is no tumor cell injection process, the destruction of the BBB and the associated inflammatory phenomena can be avoided 227. Additionally, unlike xenograft models, mice with an intact immune system can be used, making it possible to study the effects of immune cells or immunotherapy within the tumor environment 225. Another advantage is that specific genes, such as P53 or PTEN, can be manipulated, allowing for in-depth studies of the role these genes play in tumorigenesis and treatment 275. These clear advantages make it a useful *in vivo* model to investigate tumor-intrinsic and extrinsic factors in GBM from various perspectives.

GEMMs have been successfully used in various fields such as tumor microenvironmental impact analysis, tumor candidate gene and drug validation, therapeutic efficacy evaluation, and drug resistance mechanism analysis 276-278, and have been frequently used in GBM research, especially for evaluating therapies targeting genetic mutations 275, 279, 280. In particular, GEMMs have been used to evaluate specific gene mutations and subsequent abnormal expression of related downstream signaling pathways in GBM. Overexpression of the common EGFR mutation (EGFRvIII) in GBM has been demonstrated to enhance high-grade gliomas and is associated with poor patient prognosis 271, 281, and additionally, the impact of PTEN inactivation on the development of high-grade gliomas was also evaluated 281. A particularly clinically relevant finding is that IDH1 mutations are present at a low rate in primary GBM but at a high rate in secondary GBM and are also associated with increased survival 270. Taken together, it has been demonstrated that EGFR amplification, PTEN loss, and IDH1 wild type are associated with primary GBM. Apart from this, GBM GEMMs were constructed by knocking out the tumor suppressor genes such as PTCH1, TP53, and NF1 282, and it was reported that activation of the RAS pathway due to loss of TP53 and NF1 in CNS cells can cause malignant astrocytoma formation 283.

However, GEMMs also have disadvantages. First, tumor formation is often slow, which is time-consuming and expensive. In addition, unlike direct intracranial injection, GEMM tumors can occur in various locations within the brain, not at the desired location, which may limit the evaluation of some delivery methods that require precise location 284. Another major disadvantage is that intra-tumoral heterogeneity may often be lacking compared to human GBM 227, 277.

Although the advantages of the mouse as an *in vivo* model are well established and have become the primary preclinical animal cancer model, its disadvantages are also evident, as described in this section. In addition to the disadvantages described for each mouse model, there are limitations due to significant differences between mice and humans. In particular, the mouse brain is lissencephalic, a key anatomical difference from the human brain, which results in a lack of brain gyration and cortical development 285, 286. Besides, because the lifespan of mice is dozens of times shorter than that of humans, the risk of oncogenic mutations is lower due to fewer cell divisions. Significant differences between these preclinical animal models and humans lead to the failure of therapeutic clinical trials for GBM. Therefore, the need for animal cancer models that faithfully and reliably reproduce human cancer is steadily increasing.

### Canine models

One of the conditions for an ideal preclinical model for human brain tumors is a tumor that arises spontaneously and grows within the parenchyma 287. Canine models are mammals that frequently develop spontaneous brain tumors, and the characteristics of the tumors are similar to those in humans 288, 289. A canine model in which brain tumors were induced by transplantation of tumor cells has also been reported, but it was used for purposes such as assessing the feasibility of CT-based estimation of tumor growth or training in tumor surgery rather than for studying anticancer therapy 288. Research to explore anticancer therapies has largely been conducted using spontaneously occurring canine populations.

Unlike mouse models, canine GBMs exhibit endothelial proliferation, which is a major characteristic of human GBM and may be helpful in studying therapeutic strategies targeting endothelial cell proliferation 229, 289. The detailed characteristics of canine gliomas include histological patterns including endothelial proliferation, GFAP expression, palisade necrosis, microinvasion, and hypercellular inflammation 229, 290. It also showed similarities to human gliomas by showing neural progenitor markers such as vimentin and nestin and sphere-forming ability, and were recorded to have significantly higher expression of VEGF, VEGFR-1/2, EGFR-1, and PDGFα 291, 292. A study was conducted to characterize the mutational process by comparing the molecular profiles between canine gliomas and human pediatric and adult gliomas. As a result, they showed alterations found in human gliomas such as TP53 and cell cycle pathways, receptor tyrosine kinases, and IDH1 R132, and canine gliomas showed high similarity to human pediatric gliomas based on the timing of mutations and DNA methylation patterns 293. Another advantage of the canine model is that it can mimic the heterogeneity seen in human GBM patients. Canine gliomas occur across a wide range of age groups and breeds and show substantial heterogeneity, with different tumor types and grades, including astrocytomas, arising in distinct brain regions and exhibiting diverse immunological and genetic profiles 294, 295. Therefore, the canine model may be a good preclinical model for GBM research.

The canine models have been proven and used in practice to be suitable models for studying human brain tumors, showing high similarity to humans 296, 297. The similarity between the canine model and human gliomas has also been assessed from an immunological perspective. Glioma-associated microglia/GAMs are known to influence glioma progression and are polarized toward a tumor-supporting phenotype by TGF-β. In spontaneous canine gliomas, GAMs diffusely infiltrated canine astrocytomas, which correlated with an increased inflammatory cytokine environment, including TGF-β1. In addition, TGF-β1 was observed to promote tumor cell migration *in vitro*. This suggests that the GAM profile of high-grade canine astrocytomas is similar in many respects to that of adult high-grade gliomas 298. Based on these similarities, it has been used in studies of anticancer drug delivery methods. The conjugation of doxorubicin to non-living bacteria-derived minicells and delivery to tumor cells via EGFR targeting was evaluated using a spontaneous canine brain tumor model 299. The doxorubicin-loaded minicells were shown to be able to cross the BBB and selectively inhibit glioma cells while avoiding systemic toxicity, indicating strong potential for clinical application for the effective treatment of patients with brain cancer, leading to a phase 1 human clinical trial 300.

However, the high cost and ethical issues associated with using canines as animal models are major disadvantages and limitations 301. Additionally, while spontaneous canine gliomas are useful for testing new treatments, they are not suitable for studying the effects of single mutations, for which genetic GEMMs are preferred 294. Another limitation is the diversity of genetic composition. Crossbred canines with mixed backgrounds better mimic the diverse genetic background characteristics of humans, whereas inbred canines often lack genetic diversity 302.

### Non-human primate models

Non-human primates (NHPs) are phylogenetically closest to humans, providing significantly higher genetic, physiological and anatomical similarities. Therefore, they can most closely reproduce actual human diseases and expected responses to treatment 303. However, primary tumors in NHPs are rare, and transplantation using tumor cell lines from other animals is limited by immune rejection 304. As a result, there are reports of cases that cause glioma in NHPs. It was demonstrated that treatment of Macaca mulatta (Rhesus monkeys) with fractionated whole-brain radiotherapy for 2 weeks resulted in the development of gliomas in most M. mulatta, which were histologically and genomically similar to human gliomas 305. However, the period from radiotherapy to onset varied between 2.9 and 8.3 years, and this long latency may limit its application in clinical trials.

The NHP model may be an attractive preclinical model for successful clinical trials due to its advantage in best mimicking human physiology, but its practical use has been limited due to ethical concerns and prohibitive costs 306. In particular, the lack of specialized experimental equipment required for genetic modification of large animal models makes it difficult to induce tumors in NHPs 307, 308. Nevertheless, NHP models are being used to evaluate novel approaches for the treatment of gliomas. Carboplatin chemotherapy has shown limited clinical efficacy, which was thought to be due to its low tissue concentration when administered intravenously 309. Therefore, ultrasound-induced BBB disruption was evaluated using a primate model to improve the delivery of intravenously administered carboplatin to brain tissue 310. The results demonstrated that BBB opening using an implantable ultrasound transducer improves the distribution of carboplatin locally in the brain, leading to a phase 1 clinical trial.

The biggest limitations of the NHP model are ethical issues and enormous costs. There is a debate about whether studies using NHPs are necessary and justifiable, which has led to a decline in the use of NHPs in the United States and the European Union 311, 312.

### Other animal models

Porcine models are another large animal model that can be used for cancer research. Minipigs of the Yucatan, Göttingen and Sinclair breeds grow up to 35-90 kg, which is similar to the weight of a human 313. Previous studies have provided genome sequences of several laboratory pig breeds 314, 315, and comparative analyses of pig and human genomes have revealed similarities in epigenetic regulation and gene transcription profiles, which has had a major impact on the development of human genetic disease modeling 316, 317. The pig brain is anatomically very similar to the human cortex, and can reproduce tumor infiltration, drug administration, and diffusion within the cortical region 313. However, tumor formation is rare in pigs, and there is a lack of understanding of natural tumor formation in pigs due to the limited lifespan of livestock breeds prior to commercial exploitation 318. Therefore, there is no evidence for spontaneous glioma formation in pigs, and pig models have been developed by inducing gliomas.

In 2013, the first pig model of glioma using human CDX was developed 319. Landrace pigs, a domestic breed, were used, and U87MG cell lines were transplanted at 3 months of age. To overcome immune rejection, sufficient immunosuppression was performed using oral cyclosporine. Of the 15 pigs transplanted with U87MG, 14 showed macroscopic tumor formation and neurological symptoms within 30 days, and histopathological analysis showed increased cellularity, normal brain parenchyma infiltration, pseudo-palisading necrosis, and angiogenesis. Subsequently, in 2020, the first genetically engineered pig model of glioma induced by a lentiviral vector was developed 320. Göttingen minipigs were injected with lentiviral vectors expressing PDGFB, constitutively active HRAS, and shRNA-p53. As a result, histopathological analysis revealed high-grade glioma features, including high cellularity, increased Ki-67 expression, astrocytic morphology, GFAP and oligodendrocyte transcription factor 2 (OLIG2) expression, and white and gray matter infiltration.

## Future perspectives and conclusion

GBM remains one of the most formidable challenges in modern oncology due to its aggressive nature, high therapeutic resistance, and almost inevitable recurrence. Despite incremental advances in surgical techniques, radiation protocols, and chemotherapeutic agents, the median survival for GBM patients remains unacceptably short, underscoring a critical need for innovative strategies that can overcome the inherent complexities of this malignancy. Traditional 2D *in vitro* cell cultures, while valuable, often fail to adequately recapitulate the intricate cellular and molecular dynamics of the GBM TME, leading to a translational gap between preclinical findings and clinical outcomes. Consequently, promising therapeutic candidates that demonstrate efficacy in simplified model systems frequently fail to translate into meaningful clinical benefit for GBM patients. However, recent advancements in GBM microfluidic chip platforms and 3D bioprinting technologies offer a compelling path forward. These sophisticated modeling systems provide unprecedented opportunities to reconstruct the complex interplay of cellular and extracellular components that define the GBM TME, including vascularized networks, hypoxic gradients, dynamic cell-cell interactions, and the heterogeneous distribution of ECM components. By incorporating patient-derived GBM cells, immune cells, and stromal cells, these advanced models can more accurately mimic the intra-tumoral heterogeneity and personalized biology that are hallmarks of GBM. Moreover, these models enable researchers to investigate the dynamic interactions between GBM cells and the TME, elucidate the mechanisms of therapeutic resistance, and screen for novel therapeutic targets and drug combinations in a physiologically relevant context. Despite the rapid advancement of GBM-on-a-chip and 3D bioprinted platforms, several translational barriers must still be overcome before these models can meaningfully reduce failure rates in GBM clinical trials 321. Key challenges include limited large-scale clinical validation linking model-predicted drug responses with patient outcomes, incomplete recapitulation of critical microenvironmental components such as functional vasculature, BBB interfaces, and immune interactions, and insufficient standardization across chip design, biomaterials, and analytical readouts 322.

In addition, integration into pharmaceutical development pipelines will require improved scalability, automation, and regulatory alignment to demonstrate added predictive value over existing preclinical models. In this context, advancing GBM-on-a-chip technologies toward high-throughput and automation-compatible formats will be essential for overcoming current translational bottlenecks. Transitioning from bespoke PDMS-based fabrication to scalable manufacturing strategies, such as injection molding of thermoplastics, offers a practical route to improving reproducibility, throughput, and compatibility with standardized pharmaceutical workflows. Together with plate-aligned microfluidic architectures that enable parallel drug and combination screening, these approaches are expected to facilitate regulatory alignment and accelerate the integration of GBM-on-a-chip platforms as predictive non-clinical testing tools 323.

To address these challenges and ultimately improve patient outcomes, future research efforts must prioritize the development and validation of advanced translational models that accurately reflect the complexities of GBM biology. These models should be used to: Identify novel therapeutic targets: By leveraging microfluidic and bioprinted models to screen for vulnerabilities within GBM cells and the TME, researchers can uncover new targets for drug development, including those that specifically target GSCs or modulate the immune response. Evaluate drug combinations: Due to the complex and multifactorial nature of GBM, combination therapies are expected to outperform single-agent treatments in terms of efficacy. Advanced models can facilitate the rapid and efficient evaluation of drug combinations, identifying synergistic interactions and optimizing treatment regimens. Personalize treatment strategies: The use of patient-derived cells in bioprinted models allows for the development of personalized treatment strategies that are tailored to the specific characteristics of each patient's tumor. This includes selecting the most effective drugs, optimizing drug dosages, and predicting treatment responses. Understand the mechanisms of resistance: By studying how GBM cells adapt and resist therapy within the TME, researchers can identify new strategies for overcoming resistance mechanisms and improving treatment outcomes. This includes investigating the role of epigenetic modifications, metabolic adaptations, and immune evasion. Together with these advanced *in vitro* models, the need for preclinical *in vivo* models with better GBM replication is emphasized to overcome the poor overall clinical predictability. As described in this review, *in vivo* models with better replication have been steadily developed for GBM mechanistic studies and effective therapeutic strategies, and this is currently in progress.

Artificial Intelligence (AI) and Machine Learning (ML) aid in optimizing complex 3D GBM models through two distinct mechanisms: constraint-driven design automation and high-dimensional phenotypic profiling. First, AI aids in optimizing chip design by automating the determination of complex geometric parameters. The design of microfluidic chips for GBM requires precise control over multiple variables, such as channel dimensions, fluidic resistance, and shear stress, to accurately mimic the vascular niche of tumor and interstitial pressure. Traditionally, this relies on iterative trial-and-error; however, recent methodologies in design automation utilize algorithms to automatically generate chip layouts that strictly satisfy these physiological constraints, reducing the barrier to adoption for non-experts 324, 325. Furthermore, advanced optimization strategies, such as ML-driven cyclic optimization and Bayesian techniques, enable the systematic exploration of vast design spaces to predict optimal configurations for desired fluid behaviors 326, 327. By automating these variables and predicting fluid dynamics such as plasma separation efficiency via Computational Fluid Dynamics (CFD) integration, AI significantly reduces the fabrication cycles required to produce physiologically relevant GBM microenvironments 328.

Second, ML aids in data interpretation by decoding the complex, non-linear relationships between cellular phenotypes and therapeutic responses. 3D GBM models generate massive datasets from high-content imaging and real-time biosensors that exceed human analytical capacity. Deep learning algorithms facilitate the interpretation of these data by performing morphological profiling, identifying subtle structural features in GBM spheroids, such as changes in nuclear texture or invasive edge morphology, that serve as early indicators of drug efficacy 329. For instance, ML-based analysis has been shown to accurately classify drug mechanisms of action and predict concentration-dependent cytotoxicity in U87 GBM spheroids, outperforming traditional viability assays 330. Additionally, AI-driven analytics can integrate these imaging features with multi-omics data and real-time sensing signals to predict patient-specific drug resistance pathways and monitor metabolic states 331, 332. Collectively, these AI-driven capabilities transform GBM-on-a-chip from simple culture platforms into predictive precision medicine tools.

An emerging direction in cancer immunology and neuro-oncology is the establishment and utilization of PDXs in humanized mice with a human immune system 333. As described previously, PDX has the advantage of being able to reproduce human GBM most closely with regard to histology and intra-tumoral heterogeneity. However, the main limitation is that immune-related studies cannot be performed because immunodeficient mice are used. The immune system plays an essential role in GBM treatment 334, and the inability to conduct studies such as immunotherapy that actively intervene in tumor killing is a significant limitation. Given the limitations of syngeneic and xenograft models or GEMMs in reproducing the histological and genetic characteristics, intra-tumoral heterogeneity, and immune system of human cancer, the need for humanized models is clear. Accordingly, a humanized cancer mouse model co-transplanted with human tumor and immune cells into NOD-SCID IL2R-γ null mice has been developed and is being used for immuno-oncology research with potential for clinical translation 335. However, humanized mice are still limited in terms of modeling human immunity, especially in antibody response capacity 336. Successful humanized immune reconstitution mouse models with continuous improvement are expected to increase the possibility of establishing a personalized approach to implanting patients' tumors to contribute to therapeutic decision-making.

CNS tumors are classified into several subtypes according to criteria selected by WHO 337. In particular, the most important factor in classifying diffuse glioma as GBM is IDH status. IDH-wild type is classified as GBM, mutant type is classified as astrocytoma and oligodendroglioma, and IDH-mutant glioma is subdivided according to histologic grade and molecular profiles 337. IDH mutant glioma is known to predict a better prognosis due to its longer overall survival compared to wild type 338.

However, IDH-mutant type is usually found in secondary tumors rather than primary tumors, suggesting that low-grade gliomas with IDH mutations often relapse to higher grades through malignant transformation 339. Therefore, research on various types of gliomas, including GBM, is also considered important, and in August 2024, Vorasidenib, a treatment for IDH1/IDH2-mutant glioma developed in France, was approved by the FDA, which is the new drug for glioma in 25 years 340.

Research on IDH-mutant glioma is also actively underway, and mouse models suitable for the purpose have been established. In 2015, the GL261 cell line transfected with the IDH1R132H DNA vector was transplanted into C57BL/6N mice to create an mIDH1 murine model, and peptide immunization was evaluated 341. Subsequently, in 2018, the role of IDH1R132H on glioma development was evaluated in mice using the RCAS/TVA retroviral vector system 342. Additionally, a protocol to establish IDH1-mutant astrocytoma GEMM using CRISPR/Cas9 technology and adeno-associated virus (AAV) was proposed in 2023, but it has the limitation of an incubation period of more than 8 months, and troubleshooting is required 343. In this way, research on various types of glioma as well as GBM and establishment of *in vivo* models are steadily progressing and have important implications.

In conclusion, overcoming the challenges of GBM requires a concerted effort to bridge the persistent translational gap between preclinical research and clinical application. This review emphasizes the need to move beyond technology-centric modeling toward integrated, clinically informed platforms that synergistically combine microfluidic and 3D bioprinted systems to more faithfully recapitulate GBM pathophysiology, therapeutic resistance, and patient-specific tumor heterogeneity. By embracing advanced modeling strategies that reconstruct the tumor microenvironment and enable personalized therapeutic evaluation, bioengineered GBM models can evolve from experimental research tools into clinically actionable systems for precision neuro-oncology. Ultimately, integrating these innovative platforms into clinical trials will be essential to accelerate the development of effective therapies and to transform the standard of care for patients suffering from this devastating disease.

## Figures and Tables

**Table 1:** Overview of GBM models using microfluidic and bioprinting platforms

Model Type	Cell Source	Materials	Key Features	Advantages	Limitations	Applications	Ref
Microfluidic chip	GS5, GFP-HUVECs	PDMS	• Perivascular niche modeling	• Mimics the *in vivo* microenvironment • Dynamic tumor-EC interactions	• Incomplete mimicry • Short-term observation	• Microvasculature-on-a-Chip • Drug screening	85
	U87MG	PEGDA hydrogel	• Integration of microwell arrays and microfluidic channels • 3D tumor spheroid formation	• Tunable drug release • Multi-drug administration • Massive parallel testing	• Static culture conditions • Diffusion-induced channel crosstalk	• High-throughput drug screening on 3D spheroid	86
	T98G, DRG neurons, Hippocampal neurons, Panc-1, A375, A549, MCF-7, SW620, PC-3	PDMS	• Microgroove structure • Tumor neural microenvironment	• Physical separation with functional connection • Drug diffusion control • High-throughput drug screening	• Complexity of microfabrication	• Neuron-GBM-on-a-Chip • Drug screening	87
	U251, Caco-2, HepG2	PDMS, mPES Hollow fiber, Matrigel	• Multi-organ co-culture • Gut-liver drug metabolism on GBM • Hollow fiber	• *In vivo*-like intestinal functionality • Dynamic drug delivery • Long-term culture capability	• Complexity of fabrication	• Gut-Liver-GBM on-a-chip • Prodrug metabolism studies, Drug screening	88
	GBM22, iPS-EC, Pericyte, Astrocyte	PDMS, fibrin	• BBB recapitulation • Vascularized GBM model	• *In vivo*-like BBB functionality • Quantitative 3D spatio-temporal analysis	• Absence of fluid flow • Absence of immune cells or neurons	• BBB-on-a-chip • Drug delivery across the BBB studies • Drug screening	89
	Patient-derived GBM	PDMS, ETFE	• Two bonded glass layers • Tissue chamber with microchannel network	• *Ex vivo* maintenance of human GBM tissue • Continuous tissue perfusion capability	• Short-term tissue maintenance • Limited correlation with patient outcomes • Complexity of fabrication	• Preclinical model • Personalized treatment • Drug screening	90
	Patient-derived GBM, hBMVECs, HMC3, U937	PDMS, MMP-sensitive HA	• *ex vivo* GBM model • Recapitulation of the immunosuppressive microenvironment	• Predictability of clinical outcomes • High-throughput drug screening	• Use of allogeneic immune cells • Poor recapitulation of the BBB	• GBM-on-a-chip • Patient-specific screening for immunotherapy responses	91
	Patient-derived GBM, FNS, hCMEC/D3, HUVECs, HMVEC-L	PDMS, SU-8 negative photoresist, PDL, CollagenⅠ, Collagen Ⅳ, Laminin	• Perivascular niche modeling • Serum-free culture	• Maintenance of GSC stemness • High-throughput drug screening	• Insufficient recapitulation of the GBM microenvironment	• GSC-EC interaction studies • Drug screening	92
Bioprinting	U87MG, SU3(GSC)	Gelatin, alginate, fibrin	• Cell-laden scaffold • Layering structure	• Precise control over cell density and spatial distribution • Improved cell-cell and cell-ECM interactions	• Complexity in long-term culture, Unknown reprogramming potential • Complexity of imaging and analysis	• GSCs studies • Glioma drug resistance studies • Drug screening	93
	U118	Gelatin, sodium alginate, fibrin	• Cell-laden scaffold • Layering structure • Epithelial-mesenchymal transition	• Enhanced GSC enrichment • Enhanced stemness and drug resistance	• Complexity of imaging and analysis	• GSCs studies • Epithelial-mesenchymal transition studies • Drug screening	94
	U118, HUVECs	Sodium alginate, Collagen	• Coaxial bioprinting • Cell-laden printing	• Multilayered tubular structures • Induction of angiogenesis by glioma cell	• Unclear necessity of tubular structure • Absence of fluid flow	• Tumor angiogenesis studies • Drug screening	95
	U118, GSC23	Gelatin, alginate, fibrinogen	• Coaxial bioprinting • A separable core-shell design • Cell-laden printing	• Multilayered tubular structures • Facilitated analysis	• Loss of core cells near the shell • Decreased cell viability within the shell	• Drug resistance studies • Biomarker discovery • Drug screening	96
	GSC23, MSCs	Gelatin, alginate, fibrinogen	• Coaxial bioprinting • Self-assembled heterogeneous tumor fibers • Scaffold-free tissue constructs	• Cell viability and tissue organization • Promotion of ECM secretion	• Lumen collapse during long-term culture • Difficulty in removing dead cells	• Tumor studies • Drug screening	97
	GSCs(CW468, GSC23, 2907, 3264), THP-1 derived macrophage, Human astrocytes, NPCs	GM-HA, GelMA	• DLP-based 3D bioprinting system • Including M2 macrophages • Patient-derived ECM	• High-resolution spatial placement of cells and ECM • Reflection of pathological ECM characteristics	• Phototoxicity-induced cell damage • Limited scalability	• Therapeutic response profiling • Drug screening • Precision disease model	98
	GBM (DK-MG, U251MG), MSCs	CELLINK Laminink521, CELLINK Bioink biomaterial	• GBM-MSC interaction • Chemokine pattern analysis	• Reproducible platform • Manipulation of various variables • Recapitulation of chemokine signaling patterns	• Limitations of model standardization • Simplified model (GBM-MSC interactions only)	• Cell-microenvironment signaling validation • Drug screening	99
	GBM(isocitrate dehydrogenase-wild type), HUVECs	Gelatin, collagen I	• Perfusable vascular channel • 2GMFMT(mesoscopic fluorescence molecular tomography)	• Non-invasive • Real-time 3D imaging • Long-term Viability	• Limited Scalability for High-Throughput Testing • Limited single-cell imaging resolution	• Long-term monitoring of tumor behavior and drug response • Drug screening	100
	Patient-derived GBM, U-87MG, T98G, U373, 293T, Saos-2, MDA-MB-231, hAstro, hMG, human Pericyte, GL261	Fibrin,Gelatin,Transglutaminase, Pluronic F127	• Perfusable vascular channel • Multicellular 3D co-culture	• Brain-mimetic mechanical characteristics • Long-term Viability • Standardized fabrication protocol	• Tissue collapse during long-term culture • Limited reproducibility even with standardized protocols	• Long-term monitoring of tumor behavior and drug response • Personalized therapy screening	101
Bioprinting / Microfluidic	A-172, HUVECs, hCMEC/D3	PDMS, GelMA-Alginate, GelMA-Fibrin	• Microgravity condition • Cell-ECM mechanical interaction	• Gene expression analysis • Physiological flow-controlled perfusion • High-resolution imaging of junctional proteins	• Limited Long-term Viability • Incomplete mimicry	• Pre-clinical approach in brain tumor therapy • Drug screening • Drug delivery across the BBB studies	102
	Patient-derived GBM, HUVECs	Brain dECM, Silicone ink	• Brain dECM • Oxygen gradient	• Highly biomimetic brain dECM • High heterogeneity recapitulation	• Complexity of fabrication • Inability to measure the oxygen gradient	• Patient-specific drug screening • Identification of mechanisms of therapeutic resistance	103

Abbreviations: 293T, human embryonic kidney cells; A375, malignant melanoma; A549, non-small cell lung cancer; Caco-2, human colon cancer cell line; DRG, dorsal root ganglion; ETFE, ethylene tetrafluoroethylene; FNS, foetal neural stem; GelMA, gelatin methacryloyl; GL261, mouse glioblastoma cells; GSC, glioma stem cell; HA, hyaluronic acid; HepG2, human liver cancer cells; HMC3, human microglia cell line; hAstro, human astrocyte cells; hBMVECs, human brain microvascular endothelial cells; hCMEC/D3, human brain microvascular endothelial cell line; hMG, human microglia; hPericytes, human pericytes; HMVEC-L, human lung microvascular endothelial cells; HUVEC, human umbilical vein endothelial cells; MCF-7, breast cancer; MDA-MB-231, human breast cancer cells; MMP, matrix metalloproteinase; mPES, modified polyethersulfone; MSCs, mesenchymal stem cells; PDL, poly-D-lysine; PEGDA, photo-polymerizable polyethylene glycol diacrylate; PC-3, prostate cancer; Saos-2, human osteosarcoma cells; SW620, colorectal cancer; U118, human glioblastoma cells; U251, human glioblastoma cells; U373, human glioblastoma cells; U937, human monocytic cell line.

**Table 2:** Comparative summary of *in vivo* GBM animal models: characteristics and research applications

Animal models		Subtype	Method	Key Features	Pros	Cons	Applied Study Types
**Mouse**	Syngeneic models	GL261 CT-2A SMA-560 SB28	Syngeneic cell line implantation in immuno-competent host	• Histologically similar to hGBM • Recapitulates high-grade glioma • TMZ-resistant • Expresses stem markers • Checkpoint therapy resistant	• Historically well-validated model • Applicable to resistance studies, GSC research and immunotherapy studies	• High immunogenicity • High mutational load • Less invasive than hGBM • Lacks intra-tumoral heterogeneity	• Chemotherapy Immunotherapy • Combination therapy • GSC research • TME/Cytokine analysis • Drug delivery • Vaccines • CAR-T studies
	Xenograft models	U87MG U251MG (CDX)	Xenogeneic cell line implantation in immuno-deficient host	• Genetic similarity to hGBM • Expresses CD133 • Forms tumorspheres Methylated MGMT status • High proliferation	• Historically well-validated model • Commonly used model • Applicable to GSC research • Reproducing the histopathology of hGBM	• Not suitable for immunotherapy studies • Lacks diffuse infiltration and necrosis • Phenotype drift by genetic alterations • Responsive to both radiation and TMZ • Authenticity concerns Cross-contamination issue	• Chemotherapy • Combination therapy • Natural compound studies • Resistance model studies • Apoptosis modulation • Drug delivery
		Patient- derived cell (PD)	Patient-derived cell implantation in immunosuppressed host	• Represents hGBM physiology • Requires site-specific consideration • Subtype-specific properties	• Closely mimics hGBM pathology • Orthotopic model reliably reflects primary tumor • Long-term stability	• Not suitable for immunotherapy studies • Time-consuming process • Inconsistent model engraftment rates • Loss of GSC characteristics during culture • Low reproducibility	• Combination therapy • Resistance model studies • Drug efficacy evaluation
	GEMMs	Genetically induced glioma	Viral vector (RCAS-TCA vector) CRISPR/Cas9	• Targeted gene modification (EGFR, PTEN, BRAF, TP53) • Tumor develops in a manner similar to hGBM	• High-fidelity GBM modeling • Preserves BBB integrity • Enables immunotherapy research • Enables in-depth gene function analysis in tumorigenesis and therapy	• Time- and cost-intensive due to delayed tumorigenesis • Inconsistent tumor sites restrict specific assessments • Lacks intra-tumoral heterogeneity	• Genetic pathway studies • TME analysis • Resistance mechanism studies
**Canine**		Spontaneous/ transplanted glioma	Enrollment of spontaneous cases Syngeneic cell implantation	• Histologically similar to hGBM • Exhibits endothelial proliferation • Expresses neural progenitor markers • Forms tumorspheres	• High similarity to hGBM • Enables endothelial-targeted research • Mimics intra-tumoral heterogeneity	• High cost • Ethical concerns • Not suitable for mutation-specific studies • Lack of genetic diversity	• TME research • Drug delivery • Inflammatory cytokine studies
**NHP**		Irradiation-induced glioma	Fractionated whole-brain radiotherapy	• Highest genetic, physiological, and anatomical similarity to humans • Closest phylogenetic proximity to humans	• Most accurately replicates human therapeutic responses	• Prohibitively high cost • Rare occurrence of primary tumors • Xenotransplantation limited by immune rejection	• BBB modulation • Chemotherapy • Drug delivery
**Porcine**		Transplant/ Genetically induced glioma	Xenogeneic cell line implantation in immuno-deficient host Viral vector-based gene modification	• Comparable epigenetic/gene expression patterns to humans • Anatomically similar to the human cerebral cortex	• Capable of recapitulating tumor infiltration, drug delivery, and diffusion • Capable of overcoming immune rejection	• Rare tumor formation • Limited understanding of spontaneous tumor formation	• Human CDX model • Genetically modified model
